# Computational survey of peptides derived from disulphide-bonded protein loops that may serve as mediators of protein-protein interactions

**DOI:** 10.1186/1471-2105-15-305

**Published:** 2014-09-17

**Authors:** Fergal J Duffy, Marc Devocelle, David R Croucher, Denis C Shields

**Affiliations:** School of Medicine and Medical Science, University College Dublin, Belfield, Dublin 4, Ireland; Complex and Adaptive Systems Laboratory, University College Dublin, Belfield, Dublin 4, Ireland; Conway Institute of Biomolecular and Biomedical Research, University College Dublin, Belfield, Dublin 4, Ireland; Systems Biology Ireland, University College Dublin, Belfield, Dublin 4, Ireland; Department of Chemistry, Royal College of Surgeons in Ireland, 123 St. Stephens Green, Dublin 2, Ireland

**Keywords:** Cyclic peptide, Protein loop, Protein interface, Bioactive peptide, Ribosomal cyclic peptide

## Abstract

**Background:**

Bioactive cyclic peptides derived from natural sources are well studied, particularly those derived from non-ribosomal synthetases in fungi or bacteria. Ribosomally synthesised bioactive disulphide-bonded loops represent a large, naturally enriched library of potential bioactive compounds, worthy of systematic investigation.

**Results:**

We examined the distribution of short cyclic loops on the surface of a large number of proteins, especially membrane or extracellular proteins. Available three-dimensional structures highlighted a number of disulphide-bonded loops responsible for the majority of the likely binding interactions in a variety of protein complexes, due to their location at protein-protein interfaces. We find that disulphide-bonded loops at protein-protein interfaces may, but do not necessarily, show biological activity independent of their parent protein. Examining the conservation of short disulphide bonded loops in proteins, we find a small but significant increase in conservation inside these loops compared to surrounding residues. We identify a subset of these loops that exhibit a high relative conservation, particularly among peptide hormones.

**Conclusions:**

We conclude that short disulphide-bonded loops are found in a wide variety of biological interactions. They may retain biological activity outside their parent proteins. Such structurally independent peptides may be useful as biologically active templates for the development of novel modulators of protein-protein interactions.

**Electronic supplementary material:**

The online version of this article (doi:10.1186/1471-2105-15-305) contains supplementary material, which is available to authorized users.

## Background

Cyclic peptides are macrocyclic peptides which possess where linear peptide side chains or termini are covalently bonded to shape the peptide into a ring. Macrocyclic compounds such as cyclic peptides have been a renewed focus of drug discovery in recent years [1], and identifying a biologically pre-designed set of cyclic peptides in protein sequences would be of great potential interest in pharmaceutical development. Many cyclic peptides are disulphide-cyclic, where two cysteine residues form an S-S bond between two thiol side chain groups to cyclise the peptide. Many other types of cyclic peptide are possible, including head-tail cyclised peptides, where the amino N-terminus and carboxy C-terminus are bound together with an amide bond to cyclise the peptide backbone, and other side-chain crosslinked cyclic peptides, such as those possessing amide bond between a lysine side chain and an aspartic acid side chain. Side-chain crosslinked cyclic peptides include disulphide-cyclic peptides, such as those described in this work; cyclic peptides where the side-chains mimic a peptidic bond, such as Lysine-Aspartic acid side chain cyclised peptides; as well as depsipeptides, where the side-chain crosslinking bond is an ester bond. An example cyclic depsipeptide would be a serine-aspartic acid side-chain cyclised peptide. There also exist backbone head-tail cyclic peptides with additional disulphide bonding such as the cyclotides [2] and theta-defensins [3], where the disulphide bonding serves to provide additional conformational constraint to the already cyclic peptide. Cyclic peptides are interesting from a drug development point of view, due to their generally better specificity, proteolytic resistance, and stability than linear peptides [4]. While the term "cyclic peptide" can refer to a peptide cyclised by any of the above strategies, the term is often used to refer to head-tail cyclised peptides. Therefore, this work use the term "small macrocyclic peptides" to refer to cyclic peptides in general, including the disulphide-cyclised peptides described in this work.

Many examples of bioactive and therapeutic natural peptides are known, including antibiotics [5], natural hormone mimics [6] and immunosuppresants [7]. The source of these peptides are mostly non-ribosomal plant and fungal secondary metabolites, produced by specialised non-ribosomal peptide-synthetase enzymes. These can create peptides with a wide range of unusual amino acids, that can be of varying chirality, carry modified side chains and backbones, and be cyclised [8]. Reviews by Conlon et al. [9] and Cascales et al. [10] provide more detailed overview of the diversity of small macrocyclic peptides in nature. The Cybase [11, 12] database has been developed as a publically available resource describing of sequence, structural and functional properties of naturally derived cyclic proteins and peptides.

The biological activity of naturally occurring ribosomally synthesised peptides have not been subject to any systematic surveys. Disulphide bonds are known to play a key structural role in proteins, stabilising the protein tertiary structure on a large scale, and can also influence quaternary structure via interchain disulphide bonds. However, disulphide bonds can also have a local effect in constraining a much smaller component of the structure. A protein loop is a general term for a protein secondary structural element which is not a helix or sheet, and generally exhibit a lack of hydrogen bonding and high flexibility, and often served to join other secondary structural elements. Protein turns are specific types of loops where the polypeptide chain reverses its overall direction and can be between 1 and 5 residues long (*α*, *β*, *γ*, *δ* and *π* turns) [13]. A special case of this is the *β*-hairpin turn, which connects two antiparallel *β*-sheets. These regions are known to be important in protein-protein contacts [14], and short (2-8 amino-acid) protein loops or turns can be "pinned" in place by a disulphide bond [15], forming a surface structural motif held in a relatively fixed position by the disulphide bond, thus having a certain amount of independence from the larger protein tertiary structure. This approach has previously been explored in phage-display studies [16].

The idea of finding "self-inhibitory" peptides, where a peptide derived from a protein-protein interface inhibits the formation of that interface, has been previously explored, and it has been found that many of globular protein interactions are dominated by linear peptide segments [17]. Among other applications, this approach has recently been used to identify peptides that inhibit viral membrane fusion [18]. Therefore, in principle short disulphide-bonded loops derived from protein sequences and located at protein interfaces could be synthesised separately and show a similar or related biological activity to the parent protein.

The use of small macrocyclic peptides to mimic protein loops has also been exploited for the RGD peptides [19]. The RGD tripeptide motif is a cell attachment *β*-turn motif found in numerous proteins, and small macrocyclic peptides containing this motif have been shown to inhibit integrin *αVβ*3 activity, which plays an important role in tumour metastasis.

Traditionally, protein-protein interaction inhibitors are discovered by screening compounds against a particular known "target" interaction of interest in a biochemical pathway. Our motivation in this study is to harness the vast amount of protein sequence and structural data available to develop a bioinformatic approach to identifying candidate bioactive small macrocyclic peptides from disulphide-bonded protein loops. In contrast to screening compounds against a single target, this method of analysis allows simultaneous identification of modulators against a variety of protein-protein interactions that have been evolutionarily selected for. This type of bioinformatics approach has been previously successfully used to identify peptides from signalling rich juxtamembrane regions that have the ability to modulate platelet function [20]. In this study, we surveyed the sequence, structural and conservation properties of disulphide bonded protein loops, in order to infer a set of small macrocyclic peptides capable of bioactivity outside the context of their parent protein.

## Results and discussion

### Finding disulphide-bonded loops at protein-protein interfaces

To identify short disulphide bonded loops that play a crucial role at Protein-Protein Interfaces, we set out to find known three-dimensional structures of protein complexes mediated by a disulphide bonded loops. We defined short disulphide bonded loops as those equal to or less than eleven residues in length (two flanking cysteines, plus 2-9 internal residues), and excluding cysteine-knot like regions of overlapping disulphide bridges.

**Figure 1:**
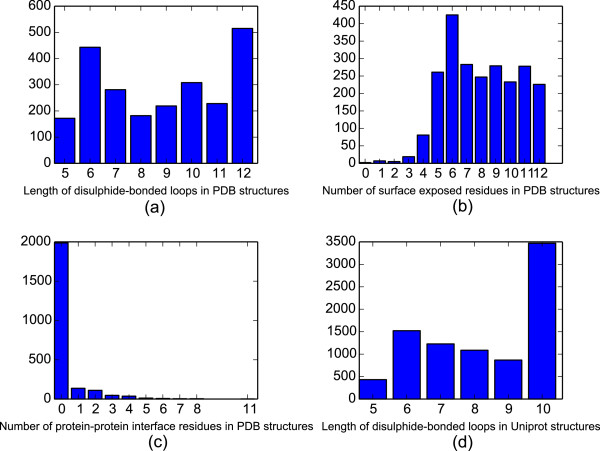
**Loop, surface and interface size distributions for disulphide-bonded loop containing proteins.** This study examined a total of 2,380 3D loop structures from 759 unique proteins in the Protein Data Bank (PDB), along with 8,607 annotated disulphide-bonded loop sequences from 5,989 Uniprot protein entries. **(a)** Shows the loop size distribution of disulphide bonded loops in PDB structures. **(b)** Shows the distribution of the number of surface residues possessed by each PDB-derived disulphide-bonded loop **(c)** shows the distribution of the number of residues in each PDB-derived disulphide loop that are involved in a protein-protein contact. **(d)** Shows the size distribution of short disulphide-bonded loops annotated in Uniprot.

2,380 Protein Data Bank (PDB) structures [21] corresponding to Uniprot entries with annotated short disulphide-bonded loops were downloaded. (Figure 1(a)) Disulphide-bonded loops at protein surfaces or interfaces were identified as described in the Methods. The number of surface residues (defined as those having at least 2.5Å ^2^ of solvent exposed surface area), and interface residues (those within 3Å of another protein chain) were counted using PyMol [22]. After removing redundant PDB structures, we found 1,231 short disulphide-bonded loops at protein surfaces (Figure 1(b)), and 132 short disulphide-bonded loops located at known protein-protein interfaces (Figure 1(c)). Of these 132 disulphide-bonded loops, 13 occupied over 50% of the interface surface area. We also found that the number of protein-protein interface hotspots contained in these disulphide-bonded loops, as predicted by the HotRegion [23] server, is generally proportional to the interface area covered by the loops (Additional file 1: Figure S1). Thus, the majority of disulphide-bonded loops at protein-protein interfaces do not constitute the major determinant of the interaction. Nevertheless, they may still play key roles in binding and represent potential templates for mimetics that target protein-protein interactions.

The proteins containing short disulphide-bonded loops shown in Table 1, are generally membrane, secreted, or extracellular, with exceptions being palmitoyl-protein thioesterase 1, in the lysosome. The reason for this is likely that the disulphide bonds would not last in the reducing environment of the cytosol. Disulphide bonds can exist in the cytosol, but they are generally either embedded in the hydrophobic core of the protein, protected from the cytosol, or in the presence of sulfhydryl oxidases [24].

**Table 1:** **Uniprot Proteins containing a disulphide-bonded loop comprising over 50% of a PDB protein-protein interface**

Uniprot	Protein containing	Interacting	PDB ID
accession	disulphide-bonded loop	protein	
P02462	Collagen alpha-1(IV) chain [Cleaved into: Arresten],*Homo sapiens*	Homodimer	1LI1
Q7T1K6	Natrin-1 (Cysteine-rich venom protein 1) (NA-CRVP1) (Protein G2a),*Naja atra*	Homodimer	1XTA
P01133	Pro-epidermal growth factor (EGF) [Cleaved into: Epidermal growth factor (Urogastrone)], *Homo sapiens*	EGF Receptor (ErbB1), *Homo sapiens*	1IVO
P01058	Bowman-Birk type proteinase inhibitor,*Phaseolus angularis*	Bovine trypsin	1TAB
P01062	Bowman-Birk type trypsin inhibitor, *Vigna radiata*	Trypsin, *Bos taurus*	1G9I
P17734	Bowman-Birk type seed trypsin and chymotrypsin inhibitor (BTCI), *Vigna unguiculata*	Trypsin, *Bos taurus*	2G81
P0AD59	Inhibitor of vertebrate lysozyme, *Escherichia coli*	Lysosome C, *Gallus gallus*	1GPQ
Q9JHF9	Lymphocyte antigen 96 (Ly-96) (ESOP-1) (Protein MD-2), *Mus musculus*	Toll-like receptor 4 (TLR-4), *Mus musculus*	2Z64
A0S865	Irditoxin subunit B (IrTxB),*Boiga irregularis*	Iridotoxin subunit A	2H7Z
P50897	Palmitoyl-protein thioesterase 1 (PPT-1) (EC 3.1.2.22) (Palmitoyl-protein hydrolase 1), *Homo sapiens*	Homodimer	3GRO
P04004	Somatomedin-B subunit of Vitronectin (VN), *Homo sapiens*	Plasminogen activator inhibitor-1 (PAI-1),*Homo sapiens*	1OC0
P04004	Somatomedin-B subunit of Vitronectin (VN), *Homo sapiens*	Urokinase plasminogen activator surface receptor (uPAR), *Homo sapiens*	3BT2
Q03405	Urokinase plasminogen activator surface receptor (uPAR), *Homo sapiens*	Urokinase-type plasminogen activator (uPA), *Homo sapiens*	3BT2
Q8RS40	Beta-1,3-xylanase (txyA), *Alcaligenes sp.*	Homodimer	2COV

Table 1 identifies the 13 proteins containing a short disulphide-bonded loop comprising over 50% of the surface area of a PDB protein-protein interface, along with the partner protein at the interface. These interfaces come from a variety of species, including bacteria (*Alcaligenes sp.*, *E. coli*), plants (*P. angularis*, *V. radiata*, *V. unguiculata*), and animals (*H. sapiens*, *M. musculus*, *N. atra*, *B. irregularis*). They also cover a variety of protein types, including snake venoms, proteinase inhibitors, collagen, and extracellular proteins involved in growth and cell adhesion.

#### Structural independence of short disulphide loops

In order to explore the structural independence of short disulphide bonded loops when removed from their parent protein, structural models of the short disulphide loops described in Table 2 were generated using the PEP-FOLD [25]*de-novo* peptide structure prediction webserver. These models were generated from the sequence of the disulphide loop alone. Five PEP-FOLD model structures were generated for each disulphide bonded loop in Table 2. The PyMol [22] align tool was then used to align each model disulphide loop to the PDB crystal structure based on backbone C *α* atoms, and calculate an RMSD between the crystal structure and model. The complete results are shown in Additional file 1: Table S1.

**Table 2:** **Protein families containing preferentially conserved disulphide-bonded loop**

Protein family	Disulphide-bonded
	loop count
Somatotropin	91
Prolactin	50
Polygalacturonase/Endopolygalacturonase	50
Guanylate cyclase activator/Guanylin	13
Urotensin	12
Disintegrin	8
Calcitonin	6
Other	57
Total	287

(Conservation difference between disulphide-bonded loop and juxtapeptide region of over 0.30.).

The mean RMSD of the lowest energy PEP-FOLD model to each corresponding crystal structure was 2.468 ±0.767Å. As a point of reference, the ongoing Critical Assessment of Techniques of Protein Structure Prediction (CASP) experiment describes the generation of a homology structure with an accuracy of better than 6.5 Å as "not trivial", and models with an accuracy of 1.5 Å as "high-resolution". Models with an RMSD of 4 Å can be considered as having a broadly correct fold [26]. Using these values as a guideline it seems that disulphide-bonded loop structure prediction using the loop sequence alone is sufficient to predict a moderately accurate structure, lending support to the idea that these loops have a large degree of structural independence from their parent protein.

#### Short disulphide-bonded loop mediated interfaces

For many of the heterodimeric interfaces listed in Table 1, there is additional experimental evidence to suggest that the short disulphide-bonded loops play a key role in binding. In the case of the interaction of Egg Lysosyme and Inhibitor of Vertebrate Lysosyme (Ivy), a cyclic loop on the surface of Ivy (CKPHDC) has been shown to be essential for its inhibitory effect, as shown by mutagenesis studies [27]. This loop is strictly conserved across 30 members of the Ivy family, and 5 other members contain a related CExxDxC motif.

For the Lymphocyte antigen 96 and Toll-like receptor 4 interaction, a disulphide-bonded loop CHGHDDDYSFC sits on the conserved "A" patch of Lymphocyte antigen 96. Mutations of five separate amino acids in this peptide have been shown to disrupt binding, including both cysteine residues, implying that the cyclic nature of the peptide is important for complex formation [28].

A CSYYQSC disulphide-bonded loop contained within Vitronectin is located at the interface of two protein-protein complexes, those of Vitronectin with Plasminogen activator inhibitor-1 and Vitronectin with Urokinase plasminogen activator receptor. The relevance of the short disulphide-bonded loop to the interaction of Vitronectin and Plasminogen activator inhibitor-1 has been experimentally verified. Alanine scanning shows that deletion of Asp22, Glu23, Leu24, Tyr27 and Tyr28 significantly reduces binding affinity. The cyclic region of the interface covers the Cys25 to Cys31 region, including 2 of the 5 critical residues [29]. The same Vitronectin disulphide-bonded loop is critical in the binding of Vitronectin to urokinase plasminogen activator receptor. The four serine (Ser26, Ser30) and tyrosine (Tyr27, Tyr28) residues in the disulphide-bonded loop fit into a cavity on Urokinase plasminogen activator receptor that shows a high degree of shape and charge complementarity [30]. Notably, the same disulphide-bonded loop tyrosine residues are important in Vitronectin binding to both Urokinase plasminogen activator receptor and Plasminogen activator inhibitor-1. Also, a Urokinase plasminogen activator receptor disulphide-bonded loop, CKTNGDC, is involved in the interaction of Urokinase plasminogen activator receptor with Urokinase plasminogen activator, in direct contact with the Urokinase plasminogen activator Kringle domain. However, this Urokinase plasminogen activator receptor disulphide-bonded loop is not located close to the Vitronectin binding region [30]. The three proteins Urokinase plasminogen activator receptor, Urokinase plasminogen activator and Vitronectin play important roles in regulating the proteolytic degradation of the extracellular matrix and blood clots [31]. This network can also play a role in cancer progression, including degrading the extracellular matrix to facilitate cancer metastasis [32]. Thus a small macrocyclic peptide that can interfere with this network would be of clinical interest.

The Epidermal growth factor disulphide-bonded loop CVVGYIGERC interacts with domain I and III of the Epidermal growth factor receptor [33]. The position of the disulphide-bonded loop region at the interface is evident in Additional file 1: Figure S2, but there is no evidence whether this disulphide-bonded loop may have independent activity. The Epidermal growth factor receptor is part of the ErbB family of receptor tyrosine kinases which act as receptors for a variety of different Epidermal growth factor-domain containing growth factor ligands. All of these Epidermal growth factor domains contain homologous C-terminal disulphide-bonded loops.

Along with their role at the binding interfaces between different proteins, it can be seen from Table 1 that disulphide-bonded loops are also involved in interactions between protein subunits (Natrin-1, Iridotoxin), and between homodimeric interfaces (Collagen *α*-1(IV) homodimer, Palmitoyl-protein thioesterase homodimer).

For the cases discussed above (Inhibitor of Vertebrate Lysosyme, Lymphocyte antigen 96, Vitronectin, Epidermal growth factor), the disulphide-bonded loop contains all or part of the protein region responsible for the interaction, and it would be interesting to determine if the disulphide-bonded loop portion of the interface alone is sufficient to modulate the protein-protein interaction.

#### A disulphide-bonded loop region in Bowman Birk inhibitors possesses independent activity

For one of the interfaces in Table 1 it has been previously shown that the short disulphide-bonded loop portion of the interface is not only a critical part of the interaction, but can act independently of the parent protein. This is the Bowman-Birk family of serine proteinase inhibitors, where the a single disulphide-bonded loop in the protein can act as an inhibitor of trypsin at nanomolar concentrations. The active disulphide loop of the Bowman-Birk inhibitor is nine residues, and Luckett et al. [34] have identified a natural sunflower cyclic Bowman-Birk inhibitor 14 residues long (SFTI-1).

The inhibitory abilities of the disulphide-bonded loop alone has been demonstrated by Domingo et al. [35], who designed a set of eleven residue small macrocyclic peptide loops based on this loop from a variety of Bowman-Birk serine protease inhibitors, and showed that the resulting small macrocyclic peptides inhibit a similar set of serine proteases as the parent protein. The native Bowman-Birk proteins inhibit at picomolar concentrations, and the disulphide-bonded loops at nanomolar concentrations - which is the range that would be expected from a druglike molecule. This result is promising from the point of view of using these loops as lead peptides for drug discovery efforts.

#### EGF domain small macrocyclic peptides do not show independent activity

To investigate if human disulphide-bonded loops at PDB interfaces can have biological activity when extracted from their parent protein, we tested whether the EGF disulphide-bonded loop identified in Table 1 along with a panel of 14 homologous disulphide-bonded loops from related EGF-domains (Table 3) could modulate EGF receptor behaviour. These sequences were chosen to represent well-described ErbB agonists [36]: a closely related set of growth factors along with a sampling of other diverse EGF domains. The disulphide-bonded loops listed in Table 3 are mostly derived from a closely related set of growth factors in the ErbB pathway, along with a sampling of other diverse EGF domains. Thus, from a single candidate disulphide-bonded loop, a library of related disulphide-bonded loops generated during evolution can be assessed for potential biologic activity.

**Table 3:** **Cyclic peptides derived from EGF-domain containing proteins and tested for EGF activation/inhibition**

No.	Cyclic peptide sequence	Human parent protein
1	CVVGYIGERC	EGF, Epidermal Growth Factor*
2	CHSGYVGARC	TGFA, Transforming Growth
		Factor Alpha*
3	CQQEYFGERC	AREG, Amphiregulin*
4	CDEGYIGARC	BTC, Betacellulin*
5	CHPGYHGERC	HBEGF, Heparin-binding EGF-like
		growth factor*
6	CEVGYTGVRC	EREG, Epiregulin*
7	CQPGFTGARC	NRG1, Neuregulin-1**
8	CPNGFFGQRC	NRG2, Neuregulin-2**
9	CKEGYQGVRC	NRG3, Neuregulin-3**
10	CVENYTGARC	NRG4, Neuregulin-4**
11	CAQECVHGRC	PEAR1, Platelet endothelial
		aggregation receptor 1
12	CTRTGYSGPNC	PTGS1, Prostaglandin G/H
		synthase 1
13	CDPGFSGLKC	SELE, E-selectin
14	CLPAFEGRNC	F7, Coagulation factor VII
15	CDSDWTGYYC	ITGB3, Integrin Beta 3

^*^Denotes growth factors known to directly activate the EGF receptor. ^**^Denotes growth factors known to directly activate members of the ErbB receptor family other than EGFR/ErbB1.

These loop-derived small macrocyclic peptides were synthesised separately and tested for their ability to 1) activate the EGF receptor and 2) competitively inhibit the EGF receptor in the presence of native EGF. Western blotting was used to assess the amount of phosphorylated EGFR after treatment with EGF, small macrocyclic peptide, or EGF following incubation with small macrocyclic peptide, as a proxy for activation. However none of the selected peptides demonstrated any ability to either activate or inhibit the EGF receptor (Additional file 1: Figure S3). We conclude that these peptides do not show significant biological activity independent of their parent protein, in contrast with the Bowman-Birk protease inhibitor peptides.

It is possible that the disulphide loop takes a significantly different shape when removed from the context of the wider EGF protein, hence explaining the lack of biological activity observed. Additional file 1: Table S1 shows that the lowest energy *de-novo* model of this loop has an RMSD of 2.374 Å based on the C *α* alignment. This suggests that the free peptide retains a structure reasonably close to what has been seen in the crystal structure.

To explain why these EGF peptides do not have activity, we examined the structure of the EGF-EGFR complex. (PDB ID: 1IVO). The EGFR protein comprises three structural domains (I, II, and III). EGF activates EGFR by binding to a cavity between EGFR domain I and III, with binding sites existing on both domain I and III [33]. The CVVGYIGERC loop (Cys33 - Cys41 of EGF) tested here comprises a large portion of the total EGF-Domain I interface contacts in the crystal structure, but only a small proportion of the EGF-Domain III contacts (Additional file 1: Figure S2). Residues in the C-terminal end of EGF, such as Leu47 are known to make important contacts with Domain III. Thus, despite comprising a large portion of the interface, the disulphide loop is not able to fill the EGFR cavity on both sides, which would likely explain why the disulphide bonded loop is not able to conformationally shift EGFR to its active position. It is possible that the disulphide bonded loop is binding to Domain I of EGFR, but clearly any potential binding is not strong enough to compete with EGF binding to its native receptor.

### Conservation of disulphide-bonded loops

The cyclic-peptide mediated interfaces above represent an interesting set of compounds, but it is also of interest to see if disulphide-bonded loops represent a widely used natural strategy to influence protein-protein interactions, by examining evolutionary conservation of short disulphide-bonded loops in proteins.

**Figure 2:**
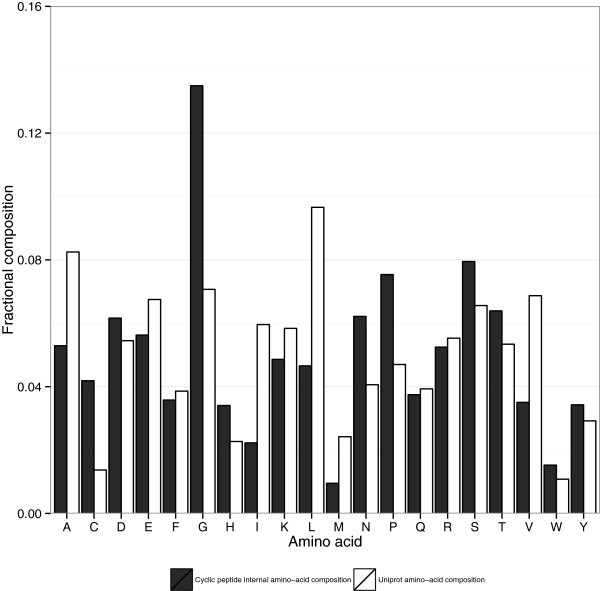
**Amino-acid distribution for proteins containing short disulphide-bonded loops.** White bars indicate fractional amino acid frequencies across all Uniprot proteins and black bars indicate amino acid frequencies inside short disulphide-bonded loops, excluding the disulphide-bond forming cysteines.

A dataset of short disulphide-bonded loop containing proteins was assembled from the SwissProt database of manually annotated proteins. Searching for all SwissProt proteins containing short disulphide bonded loops (annotated intrachain disulphide bonds with 2-8 internal residues) revealed 8607 annotated short disulphide-bonded loops in 5989 proteins (Figure 1(d) shows the size distribution of these loops). Figure 2 illustrates the distribution of amino acids in short disulphide-bonded loops, as compared to that of the full range of proteins in Uniprot. Short disulphide-bonded loops seem to contain fewer hydrophobic residues (Valine, Leucine, Isoleucine, Alanine, Methionine) which could indicate that disulphide-bonded loop loops are relatively unlikely to be located at the hydrophobic core of a protein. There is also an enrichment in Glycine and Proline residues, which are known to enable protein backbone flexibility [37], and break up helical structures [38], which may enable turns, helping the cycle to be formed.

Homologs of SwissProt proteins containing annotated short disulphide-bonded loops were identified using the Gopher [39] webserver (bioware.ucd.ie), searching the default set of model organisms. All short disulphide-bonded loop containing proteins with at least one Gopher-identified ortholog were then aligned using MUSCLE [40]. Per-residue conservation scores were then calculated for each alignment using the Jensen-Shannon divergence method of Capra and Singh [41]. Aligned short disulphide regions between the original protein and homolog were identified by examining alignments of the annotated disulphide regions of the original protein. If the loop terminal cysteine residues in the original protein exactly aligned with cysteine residues in the homolog protein, this region was considered a conserved disulphide loop. It is well known that cysteines involved in disulphide bonding are well conserved across a variety of protein families [42]. For this reason, only the conservation of the interior loop residues was considered in this study.

**Figure 3:**
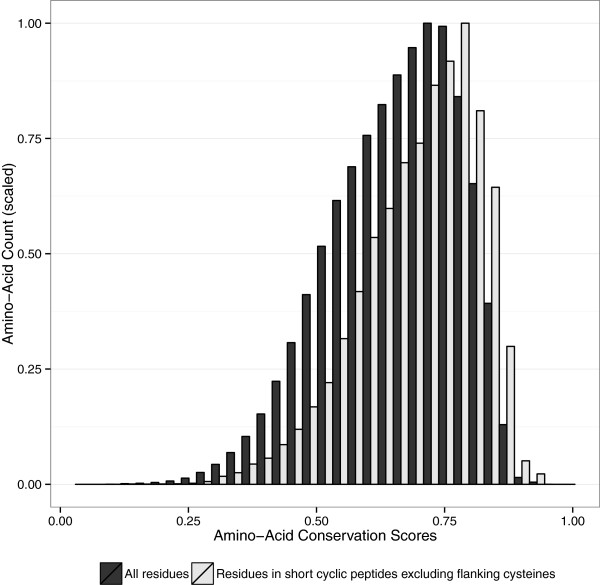
**Conservation score distribution of amino acids in proteins containing short disulphide-bonded loops.** Amino-acids inside short disulphide-bonded loops are indicated in light grey and the overall distribution is in black. Both distributions are scaled to a maximum Y-axis value of 1. P-value of <10^-15^ indicates that average conservation inside disulphide-bonded loops is very significantly larger than the overall average conservation, based on a single-tailed Mann-Whitney U test.

**Figure 4:**
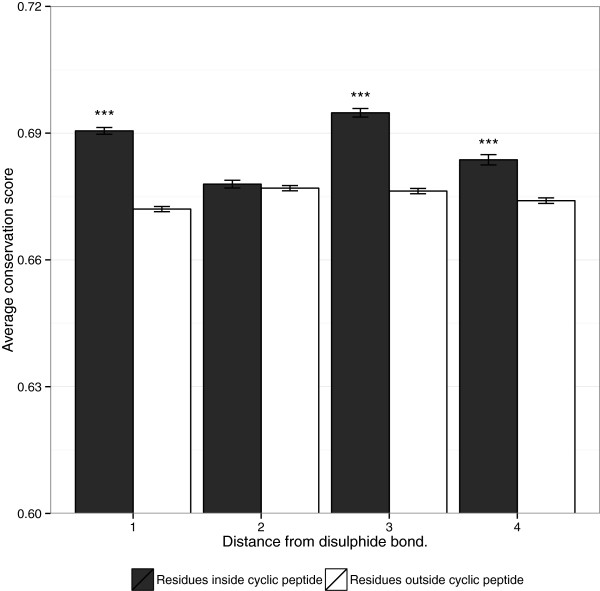
**Average amino acid conservation scores close to short loop disulphide bonds.** Error-bars indicate standard error. "***" indicates significant results, with p-values < 0.001 based on a Mann-Whitney U-test, where residues at an equal distance from a disulphide bonded cysteine are more conserved inside the disulphide bond.

**Figure 5:**
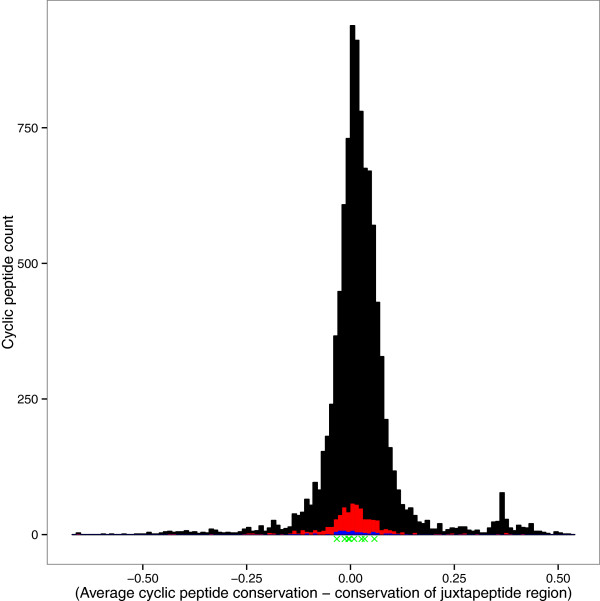
**Distribution of the differences between average disulphide-bonded loop conservation scores and the average conservation scores of residues located immediately outside the disulphide-bonded loop.** Positive values indicate disulphide-bonded loops more conserved than the regions surrounding them. The black distribution represents all SwissProt annotated disulphide-bonded loops, the red distribution represents disulphide-bonded loops on a PDB protein model surface, and the blue distribution seen at the base of the red distribution represents disulphide-bonded loops at a PDB interface. The green ’X’s underneath the histogram represent the conservation difference of the disulphide-bonded loops in Table 1 comprising over 50% of a PDB interface.

Short disulphide-bonded loops show increased conservation relative to other regions in the same set of proteins(Figure 3). It is possible that the higher conservation of these loops come from loop sequences being dominated by turn-promoting residues such as proline and glycine, which are somewhat over-represented in disulphide bonded loops, as shown in Figure 2. However it has also been observed that residues with high solvent accessibility are more variable between homologs [43, 44] than internal residues. Since we have observed that known 3D disulphide bonded loop residues are mostly exposed on the protein surface (Figure 1(b)), this variability will tend to counteract the increased likelihood of proline and glycine residues in the loop aligning by chance.Disulphide-bonded loops demonstrate a strongly statistically significant increase in conservation relative to residues immediately adjacent to the cyclic region (Figure 4), however, this increase is very modest in scale, suggesting that disulphide-bonded loop regions may often be embedded within regions of more extended structural constraint. We noted that in our dataset, the increased conservation within disulphide-bonded loops relative to adjacent regions was limited to residues 1,3 and 4 places from the cyclic cysteines and was not seen at the position two residues away. It is not clear why this would be the case, but it is possible that residues with reducing effects may have more influence at certain distances and avoidance of these effects may impact on the pattern of conservation.It may well be that disulphide-bonded loops with stand-alone function are more likely to be conserved relative to their immediately adjacent residues. Figure 5 shows the distribution of differences in conservation between disulphide-bonded loops and their immediate surrounding residues.

The surface and interface disulphide-bonded loops show a similar distribution to other cyclic regions. Thus, simply existing at a protein surface or interface does not mean that a disulphide-bonded loop will be preferentially conserved.

However, the graph does have a smaller second peak at about 0.30, indicating a set of disulphide-bonded loops that are significantly more conserved than their adjacent residues. Table 2 lists the names of the protein families that contain these highly conserved disulphide-bonded loops. Over half are secreted short proteins and peptide hormones such as somatotropin, prolactin, guanylin and urotensin, as well as the larger polygalacturonase from plants. Many of these peptides and proteins are very short, such as guanylin (15 amino acids) and urotensin (11 amino acids), with the disulphide bonded section making up over half of the peptide length. Given the lower conservation outside the cyclic region, such proteins are good candidates for investigating the role of the cyclic regions alone.

### Viral proteins as sources of bioactive disulphide-bonded loops

It is known that viruses can rapidly co-evolve many of the same protein-protein regulatory strategies as their hosts [45]. We scanned viral proteins for disulphide-bonded loops that may mimic host disulphide-bonded loops mediating interactions. Table 4 shows virus proteins containing disulphide-bonded loops, similar to a human disulphide-bonded loop, where there is at least one human-virus protein interaction described in an interaction database.

**Table 4:** **Similar disulphide-bonded loops between human and virus**

Human	Human	Human	Virus	Virus	Virus
accession	peptide	protein name	accessions	peptide	protein name
O43506	CELQWC	ADAM20: Disintegrin and metalloproteinase domain-containing protein 20	Q1HVB5	CELGWC	Uncharacterized protein BNLF2b: Human herpesvirus 4
P22413, Q13822	CKGRC	ENPP1 and ENPP2: Ectonucleotide pyrophosphatase/phosphodiesterase family member 1 and 2	O41974	CKRRC	Immediate-early protein, 73: Murid herpesvirus 4
P04626	CSKPC	ERBB2: Receptor tyrosine-protein kinase erbB-2	P03227	CRRPC	Single stranded DNA-binding protein: Human herpesvirus 4
P00451	CRAPC	F8: Coagulation factor VIII	P03227	CRRPC	Single stranded DNA-binding protein: Human herpesvirus 4
P18564	CTTSTDSC	ITGB6: Integrin beta-6	P03254	CNSSTDSC	Early E1A 32 kDa protein: Human adenovirus 2
Q96LB9	CTVSTDC	PGLYRP3: Peptidoglycan recognition protein 3	Q77PU6	CTIRSDC	Protein U90: Human herpesvirus 6B
Q92954, P04004	CKGRC	PRG4: Proteoglycan 4 and VN: Vitronectin	O41974	CKRRC	Immediate-early protein, 73: Murid herpesvirus 4

The match of an adenoviral peptide CNSSTDSC to a human integrin *β*-6 CTTSTDSC disulphide-bonded loop was of most interest, since viral proteins exploit extracellular matrix and integrin interactions to facilitate cell adhesion and entry. The integrin CTTSTDSC loop is located in the third EGF-like repeat domain region of the extracellular portion of Integrin *β*-6. This region is known to be "masked" in the integrin’s inactive conformation, and exposed in the active conformation [46]. The human herpesvirus ssDNA binding protein binds the secreted FBLN5 protein: (Additional file 1: Table S2) the viral peptide CRRPC resembles the human ERBB2 CSKPC peptide somewhat. This similarity is relatively weak, but it is intriguing that the FBLN5 protein contains 9 EGF domains, since ERBB2 belongs to a receptor family known to bind EGF domains.

Many of the matches described in Table 4 consist of quite short disulphide loops, 5 or 6 residues long, which points to the possibility of these matches being a chance occurrence. However, as described above, there is some biological support for considering viral proteomes as representing potential sources of cyclic bioactive peptides worthy of further investigation.

### Perspectives on disulphide-bonded loops in protein-protein interactions

We would have expected to uncover more disulphide-bonded loops that play key roles in protein-protein interactions, given the importance of cyclic compounds in modulating both enzyme and protein-interactions. It is possible that the selection of small loops within larger proteins is, in general, slower to evolve to the required degree of specificity of interaction than the evolution of larger complementarities between interacting proteins. Thus, in general, proteins do not often seem to evolve structured loops as independent determinants of protein interaction. This is in contrast to eukaryotic Short Linear Motifs (SLiMs) [47] which have specifically evolved to match particular motif binding domains. Why it is that biology has decided to focus on linear motifs rather than cyclic motifs in eukaryotes is not clear. However, it is worth noting that many linear motifs, although they bind independently, typically bind with modest affinity, in a manner that suggests that modest affinity is optimal [48]. Thus, the benefit of higher affinity binding that cyclic loops might confer may not be advantageous in the context of SLiM signalling, perhaps explaining why our survey has revealed relatively few such interactors. While there are examples of such loops having independent effects from the literature, overall, it may be that mining existing cyclic loop sequences from protein interactions may not be a very fruitful strategy for discovering novel modulators of protein-protein interactions, although we must point out that our own experimental validation only considered one class of such loops.

## Conclusions

Considering a small disulphide-bonded loop as a surface-exposed loop pinned in place by a pair of cysteines, the structure of this loop could be relatively independent of the sequence of the larger protein, and mostly determined by the amino acids between the cysteines, due to the conformational constraint imposed by the disulphide bond, and the solvent exposed nature of a surface loop. Thus, a disulphide-bonded loop known to be at a protein-protein interface could on its own, potentially maintain the same binding activity as the parent protein. Since cyclic peptides are known to be more "drug-like" than linear peptides, this approach could provide a promising set of biologically-optimised lead-like molecules to attack the difficult problem of modulating protein-protein interactions [49]. We have contrasted two cases in this study, that of the Bowman-Birk protease derived small macrocyclic peptides, which are known to have independent biological activity, and compared this with a range of EGF-derived small macrocyclic peptides, which do not, showing that while ribosomally derived disulphide-bonded loops can be a promising source of bioactive macrocycles, they may not contain the necessary binding features, even when they make up a majority of a protein-protein interface. Bowman-Birk inhibitors are currently being investigated for their applications in fighting colorectal cancer, due to promising mouse results [50].

A disadvantage of re-purposing disulphide bonded loops as small macrocyclic peptides is that disulphide bonds are more easily broken than the amide bonds present in a traditional head-tail cyclic peptides. Nyugen et al. [51] have compared the serum stability of short (6 residue) antimicrobial linear peptides to the disulphide and head-tail bonded equivalents, and observed that while linear peptides will be 80% degraded in serum over the course of an hour, disulphide-bonded peptides retain over 50% of their original concentration after 2 hours, and head-tail bonded peptides retain over 70% of their original concentration after 6.5 hours. The concentration of the short depsipeptide arenastatin A in mouse serum has been observed to decline 50% in an hour [52], leading to the conclusion that head-tail bonding is the most desirable from a serum stability point of view. There are a number of well-described strategies which can be used to improve the stability and bioavailability of peptide drugs in the bloodstream [53], and these strategies, along with substituting disulphide bonds for another cyclisation method may be necessary to develop a truly "drug-like" molecule.

Despite being mainly restricted to extracellular or vesicular compartments, short disulphide-bonded loops are relatively widespread with 720 out of a total of 20,252 human proteins in the SwissProt database of manually curated proteins contain annotated disulphide-bonded loops. Disulphide-bonded loop residues are more conserved than non-cyclic residues in the same set of proteins, and are also more conserved than residues located directly beside short disulphide-bonded loops. While the conservation is modest, there is a subset of peptides which show marked conservation (see Table 2), mainly short secreted peptide hormones or chemical messengers.

This is possibly due to cyclisation by disulphide bonding being the simplest way to impose some structure on a short peptide, which may not have the size to form hydrogen-bonded secondary structural features like helices or beta-sheets. Potential disulphide-bonded loops are also seen in viral proteins, and a subset of these may play potential roles in viral adhesion and entry, or other aspects of viral biology.

This study has used the approach of mining sequence databases for putative disulphide bonded disulphide-bonded loops, conserved relative to their adjacent residues, thus generating a library of compounds of interest. Table 1 contains 13 disulphide bonded loops from well characterised interaction crystal structures, of which only the Bowman-Birk type loops have ever been tested for their independent activity prior to this study. The disulphide bonded loops of interest include not only the loops described in Table 1, but, as the PDB currently only contains structures for a tiny fraction of all possible protein-protein interactions, the 1,231 short disulphide bonded loops at protein surfaces (Additional file 1: Table S3) are also of worthy of investigation, along with the 287 highly conserved disulphide-bonded loops mentioned in Table 2 which are further described in Additional file 1: Table S4.

It is well known that small macrocyclic peptides represent a class of molecules with a wide range of biological activities, and therefore merely showing that there exist bioactive small macrocyclic peptides derived from larger proteins would not represent any furthering of scientific knowledge. Thus, the key novelty of this work is in exploring how to systematically harness sequence, structural, and evolutionary data over all well characterised proteins in order to identify bioactive proteins. This work both develops a method for identifying potentially bioactive compounds, as well as providing a list of disulphide-bonded loop-protein interaction pairs, readily synthesisable and open to medium or high throughput functional screening for binding or activity.

## Methods

### Assessing short disulphide-bonded loop structures

Protein data bank (PDB) structures containing short disulphide-bonded loops were identified by searching UNIPROT for manually curated proteins containing a non-overlapping disulphide loop with 2-9 internal residues, with a listed entry in the PDB. The biological assembly format (.pdb1) of each PDB structure was downloaded. Choosing the biological assembly format ensures that the downloaded structure is the biologically relevant form of the structure, as opposed to the crystallographic asymmetric unit, which are not always the same thing. A PyMol [22] script was used to iterate over each structure file, and test each possible pair of cysteine residues spaced up to 11 residues apart in the same protein chain to see if a disulphide bond existed, by checking whether an S-S bond of approximately 2.05 Å existed. For each short disulphide-bonded loop found, the number of surface residues were found by considering a residue with a solvent accessible surface of over 2.5 Å ^2^. The number of residues at a protein-protein interface was found by counting as an interface residue all amino acids with heavy atoms within 3Å of another protein chain.

### EGF receptor activation and competition assays

MCF10A immortalised breast epithelial cells were serum starved for 3 hours before all experiments. For activation experiments, cells were incubated with either 100 *μ*M small macrocyclic peptide or 10 nM EGF for 5 minutes. For EGF/small macrocyclic peptide competition assays, the cells were incubated with 100 *μ*M small macrocyclic peptide for 5 minutes before incubating for another 5 minutes with 1 nM EGF. EGF Receptor activation was then measured by western blotting for phosphorylated Tyrosine 1173 on EGFR, along with blotting for total EGFR and total Actin.

### Predicting short disulphide-bonded loops in viruses using protein structural information

Likely short disulphide-bonded loop locations were predicted based on sequence, secondary structure and solvent accessibility information. Secondary structure and solvent accessibility were predicted by the Porter and PaleAle servers from the Distill [54] suite of protein structural prediction servers. Portions of sequences under 11 residues were included that 1) started and ended with a cysteine, 2) contained no internal cysteine residues, 3) were in a region of a protein that was not predicted to be a *β* sheet or an *α* helix, and 4) had an average solvent accessibility of the region that was predicted to be more exposed than buried.

### Scoring similarity of short disulphide-bonded loops

The similarity of two disulphide-bonded loop sequences was found by aligning the disulphide-bonded loop sequences, excluding the flanking cysteine residues, using the Bio.pairwise2.align function from the BioPython [55] package, which implements pairwise sequence alignment using a dynamic programming algorithm, scored with the BLOSUM62 scoring matrix, and a gap opening and extension penalty of -12. (This gap penalty is three times the BLOSUM62 penalty for the most dissimilar amino-acids, in order to obtain very similar length disulphide-bonded loops). The maximum possible alignment score was calculated by aligning the viral short disulphide-bonded loop to itself, and similarity between human and viral peptides was calculated as


### Identifying viral disulphide-bonded loops similar to human proteins

Human and virus proteins containing SwissProt annotated short disulphide-bonded loop regions were identified or predicted using DISTILL as described above. To identify similar viral proteins, we set out to find 1) disulphide-bonded loops with similar sequences in human and viral proteins 2) where the viral proteins had known human interactor proteins 3) where the human proteins interacting with the viral proteins containing a short disulphide-bonded loop also interacted with the original human proteins containing the similar short disulphide-bonded loop.

Uniprot accession numbers were used to uniquely identify proteins, and pairs of Uniprot accessions parsed from the interaction files were used to identify interactions. Similarity between human and virus short disulphide-bonded loop sequences was calculated by aligning and scoring the peptides using the BLOSUM62 substitution matrix to identify similar short disulphide-bonded loops in host and virus. Virus and human short disulphide-bonded loops with a similarity of greater than 0.50 were identified, and checked for shared interactors.

To find which human and virus proteins shared interactors, the sets of known human protein interactions (including human-virus interactions) were downloaded as PSI-MITAB format text files. A text file of human binary protein interactions was downloaded from the MINT [56] database (download date: 08/Apr/2013), which was then parsed to extract human-virus interactions only. A text file of human-virus protein binary interactions was downloaded from the Virhostnet [57] database (download date: 24/Apr/2013), and text files containing human-virus binary interaction data for each of the virus species available in the BioGRID [58] database were also downloaded (download date 24/Apr/2012). All three interaction sources were combined into a single dataset and compared with the list of virus and human proteins with a similarity greater than 0.50, to identify shared interactors.

## Electronic supplementary material

Additional file 1: **Supplementary material.** Contains supplementary tables and figures referred to in the article. (PDF 1 MB)
