# Supplementary material for: Computational survey of peptides derived from disulphide-bonded protein loops that may serve as mediators of protein-protein interactions
Source: BMC Bioinformatics. 2014 Sep 17;15(1):305. doi: 10.1186/1471-2105-15-305 (PMC4262234; doi:10.1186/1471-2105-15-305)
Supplement: Supplementary file 1 — Additional file 1: Supplementary material. Contains supplementary tables and figures referred to in the article. (PDF 1 MB) [file 12859_2014_6691_MOESM1_ESM.pdf]

Additional file 1: Table S3: Disulphide loops at the surface of PDB proteins.

**PDB-ID**	**Chain-ID**	**Start**	**End**
11BA	A	65	72
11BA	B	65	72
153L	A	18	29
1A0M	A	2	8
1A0M	B	2	8
1A14	N	124	129
1A14	N	232	237
1A14	N	280	289
1A21	A	47	55
1A22	A	182	189
1A22	B	238	248
1A22	B	283	294
1A2W	A	65	72
1A2W	B	65	72
1A39	A	18	24
1A39	A	63	69
1A39	A	223	228
1A4G	A	121	126

Additional file 1: Table S3: Disulphide loops at the surface of PDB proteins.

PDB-ID	Chain-ID	Start	End
1A4G	A	230	235
1A4G	A	278	288
1A4G	B	121	126
1A4G	B	230	235
1A4G	B	278	288
1A7S	A	154	160
1A8E	A	171	177
1A8X	A	50	57
1A8X	A	478	489
1AB1	A	16	26
1ACJ	A	254	265
1ACX	A	34	43
1ACX	A	83	88
1AGM	A	262	270
1AGY	A	171	178
1AHW	C	49	57
1AHW	F	49	57
1AIV	A	171	182
1AJA	A	168	178

Additional file 1: Table S3: Disulphide loops at the surface of PDB proteins.

PDB-ID	Chain-ID	Start	End
1AJA	B	168	178
1AK0	A	80	85
1AKG	A	2	8
1AKN	A	246	257
1AKP	A	37	47
1AKP	A	88	95
1ALU	A	44	50
1ALU	A	73	83
1AM5	A	45	50
1AM5	A	206	210
1AMP	A	223	227
1AN3	A	180	188
1AN3	B	14	24
1AN3	B	53	64
1AN3	C	14	24
1AN3	C	53	64
1AO6	A	53	62
1AO6	A	90	101
1AO6	A	168	177

Additional file 1: Table S3: Disulphide loops at the surface of PDB proteins.

PDB-ID	Chain-ID	Start	End
1AO6	A	245	253
1AO6	A	278	289
1AO6	A	360	369
1AO6	A	437	448
1AO6	A	476	487
1AO6	A	558	567
1AOG	A	53	58
1AOG	B	53	58
1AOL	A	73	83
1AOL	A	178	184
1APH	A	6	11
1APY	A	41	46
1APY	C	41	46
1AQB	A	120	129
1AQH	A	328	335
1ATJ	A	44	49
1ATJ	B	44	49
1ATJ	C	44	49
1ATJ	D	44	49

Additional file 1: Table S3: Disulphide loops at the surface of PDB proteins.

PDB-ID	Chain-ID	Start	End
1ATJ	E	44	49
1ATJ	F	44	49
1ATL	A	157	164
1ATL	B	157	164
1AUK	A	161	168
1AUK	A	493	499
1AUN	A	52	62
1AUN	A	67	73
1AUN	A	134	144
1AUN	A	148	157
1AUN	A	158	163
1AUT	L	59	64
1AUT	L	80	89
1AUT	L	98	109
1AVA	C	144	148
1AVF	A	45	50
1AVF	A	208	212
1AVU	A	136	145
1AYP	A	77	88

Additional file 1: Table S3: Disulphide loops at the surface of PDB proteins.

PDB-ID	Chain-ID	Start	End
1AYP	F	77	88
1AZ7	A	7	18
1AZ7	A	53	62
1B0L	A	170	181
1B0L	A	627	632
1B12	A	170	176
1B17	A	6	11
1B1X	A	170	181
1B1X	A	625	630
1B1Z	A	87	98
1B1Z	B	87	98
1B1Z	C	87	98
1B1Z	D	87	98
1B2M	A	2	10
1B2Y	A	378	384
1B30	A	256	262
1B37	A	457	463
1B3E	A	171	177
1B3J	A	36	41

Additional file 1: Table S3: Disulphide loops at the surface of PDB proteins.

PDB-ID	Chain-ID	Start	End
1B41	B	41	52
1B41	B	53	59
1B56	A	120	127
1B5F	A	45	50
1B5F	A	206	210
1B5F	C	45	50
1B5F	C	206	210
1B6E	A	59	70
1B6E	A	61	72
1B90	A	91	99
1B9S	A	122	127
1B9S	A	231	236
1B9S	A	279	289
1BBI	A	14	22
1BBI	A	32	39
1BBI	A	41	49
1BBS	A	45	50
1BBS	A	206	210
1BCP	B	192	199

Additional file 1: Table S3: Disulphide loops at the surface of PDB proteins.

**PDB-ID**	**Chain-ID**	**Start**	**End**
1BCP	C	192	199
1BCP	D	103	109
1BCP	E	103	109
1BCP	F	92	98
1BGC	A	37	43
1BGC	A	65	75
1BGD	A	37	43
1BGD	A	65	75
1BHE	A	89	99
1BHP	A	16	25
1BHT	A	74	84
1BHT	B	374	384
1BIY	A	170	181
1BIY	A	625	630
1BJ3	A	2	13
1BJ3	B	2	13
1BJ7	A	44	48
1BK7	A	15	23
1BK7	A	168	179

Additional file 1: Table S3: Disulphide loops at the surface of PDB proteins.

**PDB-ID**	**Chain-ID**	**Start**	**End**
1BLF	A	170	181
1BLF	A	625	630
1BML	A	558	566
1BML	B	558	566
1BMO	A	55	66
1BMO	B	55	66
1BOQ	A	101	111
1BP3	A	182	189
1BP3	B	212	222
1BP3	B	251	262
1BQQ	T	1133	1138
1BQU	A	16	26
1BQU	A	54	64
1BQU	B	16	26
1BQU	B	54	64
1BR9	A	133	138
1BRP	A	120	129
1BS9	A	46	52
1BS9	A	171	178

Additional file 1: Table S3: Disulphide loops at the surface of PDB proteins.

PDB-ID	Chain-ID	Start	End
1BST	A	181	189
1BSW	A	159	164
1BTE	A	86	91
1BTE	B	86	91
1BU8	A	4	10
1BU8	A	90	101
1BU8	A	285	296
1BU8	A	299	304
1BVN	P	378	384
1BWC	A	58	63
1BX7	A	6	17
1BX7	A	11	22
1BY2	A	75	85
1BY3	A	318	329
1BY3	A	692	698
1BZI	A	130	141
1C2A	A	15	23
1C2A	A	32	39
1C2A	A	74	82

Additional file 1: Table S3: Disulphide loops at the surface of PDB proteins.

PDB-ID	Chain-ID	Start	End
1C2A	A	91	98
1C3A	A	4	15
1C3A	B	204	215
1C5M	D	22	27
1C5M	F	1	12
1C7S	A	56	66
1C7S	A	400	408
1C8D	A	490	494
1C8E	A	490	494
1C9P	B	10	21
1C9P	B	15	26
1CBG	A	202	210
1CD9	A	37	43
1CD9	A	65	75
1CD9	B	13	24
1CD9	B	59	68
1CD9	C	37	43
1CD9	C	65	75
1CD9	D	13	24

Additional file 1: Table S3: Disulphide loops at the surface of PDB proteins.

PDB-ID	Chain-ID	Start	End
1CD9	D	59	68
1CDF	A	26	37
1CDF	A	111	116
1CDG	A	43	50
1CDT	A	42	53
1CDT	A	54	59
1CDT	B	42	53
1CDT	B	54	59
1CEL	A	19	25
1CEL	A	61	67
1CEL	A	238	243
1CGT	A	43	50
1CIV	A	20	25
1CLE	A	268	277
1CLV	A	354	360
1CMS	A	47	52
1CMS	A	207	211
1CN4	A	28	38
1CN4	B	28	38

Additional file 1: Table S3: Disulphide loops at the surface of PDB proteins.

PDB-ID	Chain-ID	Start	End
1CN4	C	29	33
1CNS	A	97	105
1CP7	A	245	250
1CPB	B	152	161
1CPB	B	161	166
1CPJ	A	63	67
1CPJ	A	108	119
1CPJ	B	63	67
1CPJ	B	108	119
1CPO	A	79	87
1CPY	A	224	233
1CPY	A	262	268
1CQE	A	36	47
1CQE	A	59	69
1CQE	A	569	575
1CQE	B	36	47
1CQE	B	59	69
1CQE	B	569	575
1CRL	A	268	277

Additional file 1: Table S3: Disulphide loops at the surface of PDB proteins.

PDB-ID	Chain-ID	Start	End
1CSB	B	63	67
1CSB	B	108	119
1CTX	A	26	30
1CTX	A	45	56
1CTX	A	57	62
1CVI	A	1315	1319
1CVI	B	2315	2319
1CVI	C	3315	3319
1CVI	D	4315	4319
1CVU	A	36	47
1CVU	A	59	69
1CVU	A	569	575
1CVU	B	2036	2047
1CVU	B	2059	2069
1CVU	B	2569	2575
1CVW	H	22	27
1CVW	L	91	102
1CYG	A	40	47
1CZF	A	329	334

Additional file 1: Table S3: Disulphide loops at the surface of PDB proteins.

**PDB-ID**	**Chain-ID**	**Start**	**End**
1CZF	A	353	362
1D3E	3	211	216
1D3E	3	211	220
1D3E	3	216	220
1D3E	I	21	25
1D3E	I	65	69
1D7F	A	43	50
1D7F	B	43	50
1DDT	A	461	471
1DEO	A	88	96
1DEQ	A	48	52
1DEQ	A	164	168
1DEQ	B	200	204
1DEQ	B	200	208
1DEQ	B	204	208
1DEQ	B	208	218
1DEQ	C	135	139
1DEQ	D	48	52
1DEQ	D	164	168

Additional file 1: Table S3: Disulphide loops at the surface of PDB proteins.

PDB-ID	Chain-ID	Start	End
1DEQ	E	200	204
1DEQ	E	200	208
1DEQ	E	204	208
1DEQ	E	208	218
1DEQ	F	135	139
1DEU	A	112	118
1DIX	A	18	24
1DIX	A	177	188
1DKL	A	178	188
1DKL	A	382	391
1DMT	A	56	61
1DMT	A	233	241
1DN2	E	2	12
1DN2	F	2	12
1DNQ	A	166	175
1DNQ	A	191	199
1DNQ	A	208	216
1DNQ	A	227	236
1DNQ	A	305	309

Additional file 1: Table S3: Disulphide loops at the surface of PDB proteins.

**PDB-ID**	**Chain-ID**	**Start**	**End**
1DNR	A	482	491
1DNR	A	502	511
1DNR	A	558	567
1DNR	A	596	604
1DP4	C	423	432
1DP5	A	45	50
1DQB	A	8	17
1DQB	A	47	52
1DQB	A	56	64
1DTZ	A	170	181
1DTZ	A	625	630
1DU5	A	51	61
1DU5	A	66	72
1DU5	A	132	142
1DU5	A	146	155
1DU5	A	156	164
1DU5	B	51	61
1DU5	B	66	72
1DU5	B	132	142

Additional file 1: Table S3: Disulphide loops at the surface of PDB proteins.

PDB-ID	Chain-ID	Start	End
1DU5	B	146	155
1DU5	B	156	164
1DV8	A	153	164
1DW0	A	89	97
1DWA	M	206	214
1DX5	I	351	360
1DX5	I	390	395
1DX5	I	399	407
1DX5	I	427	437
1DX6	A	254	265
1DXL	A	45	50
1DXL	B	45	50
1DYQ	A	96	106
1DYT	A	62	71
1DZA	A	165	172
1E1E	A	210	216
1E1E	B	210	216
1E3U	A	44	51
1E3U	C	44	51

Additional file 1: Table S3: Disulphide loops at the surface of PDB proteins.

PDB-ID	Chain-ID	Start	End
1E5P	A	38	42
1E87	A	85	96
1E88	A	29	38
1EBD	A	47	52
1EBO	A	100	107
1EBO	B	100	107
1EBO	C	100	107
1EDM	B	51	62
1EDM	B	73	82
1EDM	C	51	62
1EDM	C	73	82
1EDN	A	3	11
1EH5	A	152	160
1EHO	A	251	262
1EI3	A	37	46
1EI3	A	46	50
1EI3	A	162	166
1EI3	B	70	81
1EI3	B	81	85

Additional file 1: Table S3: Disulphide loops at the surface of PDB proteins.

PDB-ID	Chain-ID	Start	End
1EI3	B	198	202
1EI3	B	198	206
1EI3	B	202	206
1EI3	B	206	216
1EI3	C	8	19
1EI3	C	9	19
1EI3	C	19	23
1EI3	C	135	139
1EI3	D	37	46
1EI3	D	46	50
1EI3	D	162	166
1EI3	E	70	81
1EI3	E	81	85
1EI3	E	198	202
1EI3	E	198	206
1EI3	E	202	206
1EI3	E	206	216
1EI3	F	8	19
1EI3	F	9	19

Additional file 1: Table S3: Disulphide loops at the surface of PDB proteins.

PDB-ID	Chain-ID	Start	End
1EI3	F	19	23
1EI3	F	135	139
1EIU	A	25	31
1EN2	A	35	39
1EN2	A	82	86
1ENF	A	82	92
1EO8	B	144	148
1EPW	A	436	445
1ERB	A	120	129
1ES7	B	240	244
1ES7	B	302	307
1ES7	D	740	744
1ES7	D	802	807
1ESL	A	122	133
1ETH	A	4	10
1ETH	A	91	102
1ETH	A	286	297
1ETH	A	300	305
1ETH	B	17	28

Additional file 1: Table S3: Disulphide loops at the surface of PDB proteins.

PDB-ID	Chain-ID	Start	End
1ETH	C	4	10
1ETH	C	91	102
1ETH	C	286	297
1ETH	C	300	305
1EW2	A	467	474
1EWK	A	432	439
1EWK	B	432	439
1EX2	A	74	79
1EX2	B	74	79
1EXP	A	261	267
1EXT	A	139	150
1EXT	A	156	162
1EXT	B	139	150
1EXT	B	156	162
1EYL	A	138	147
1F0N	A	87	92
1F2S	I	316	327
1F32	A	48	59
1F34	A	45	50

Additional file 1: Table S3: Disulphide loops at the surface of PDB proteins.

PDB-ID	Chain-ID	Start	End
1F34	A	206	210
1F34	B	48	59
1F42	A	109	120
1F45	A	109	120
1F6F	A	7	14
1F6F	A	192	197
1F6F	B	12	22
1F6F	B	51	62
1F6F	C	12	22
1F6F	C	51	62
1F6W	A	246	257
1F8U	B	41	52
1F8U	B	53	59
1F94	A	6	11
1F94	A	56	61
1FAS	A	41	52
1FAS	A	53	59
1FEA	A	51	56
1FEA	B	51	56

Additional file 1: Table S3: Disulphide loops at the surface of PDB proteins.

PDB-ID	Chain-ID	Start	End
1FF4	A	46	57
1FF4	A	58	63
1FG9	C	60	68
1FG9	C	178	183
1FG9	D	60	68
1FG9	D	178	183
1FG9	E	60	68
1FG9	E	178	183
1FJR	A	61	66
1FJR	A	71	82
1FL7	B	87	94
1FLC	A	332	338
1FLC	B	145	149
1FLC	C	332	338
1FLC	D	145	149
1FLC	E	332	338
1FLC	F	145	149
1FM5	A	85	96
1FSI	A	104	110

Additional file 1: Table S3: Disulphide loops at the surface of PDB proteins.

PDB-ID	Chain-ID	Start	End
1FSU	A	181	192
1FVU	A	2	13
1FVU	B	402	413
1FVU	C	202	213
1FVU	D	602	613
1G0X	A	134	144
1G13	A	68	75
1G13	A	94	105
1G1Q	A	122	133
1G1Q	A	144	153
1G1Q	B	122	133
1G1Q	B	144	153
1G1Q	C	122	133
1G1Q	C	144	153
1G1Q	D	122	133
1G1Q	D	144	153
1G3P	A	46	53
1G5G	A	338	347
1G5G	A	362	370

Additional file 1: Table S3: Disulphide loops at the surface of PDB proteins.

PDB-ID	Chain-ID	Start	End
1G5G	A	394	399
1G5G	B	338	347
1G5G	B	362	370
1G5G	B	394	399
1G5G	C	338	347
1G5G	C	362	370
1G5G	C	394	399
1G72	B	6	12
1G72	D	6	12
1G8T	A	9	13
1G8T	B	9	13
1G96	A	73	83
1G9I	I	303	307
1G9I	I	309	317
1G9M	G	228	239
1G9N	G	228	239
1GBS	A	18	29
1GCY	A	140	150
1GER	A	42	47

Additional file 1: Table S3: Disulphide loops at the surface of PDB proteins.

PDB-ID	Chain-ID	Start	End
1GER	B	42	47
1GF2	A	46	51
1GH7	A	11	21
1GH7	A	62	67
1GH7	A	226	236
1GH7	B	11	21
1GH7	B	62	67
1GH7	B	226	236
1GK8	A	449	459
1GK8	C	449	459
1GK8	G	449	459
1GL0	I	17	27
1GL1	I	17	28
1GL4	A	384	395
1GML	A	366	372
1GML	D	366	372
1GMY	A	63	67
1GMY	A	108	119
1GMZ	A	75	86

Additional file 1: Table S3: Disulphide loops at the surface of PDB proteins.

PDB-ID	Chain-ID	Start	End
1GOF	A	18	27
1GOK	A	255	261
1GPL	A	4	10
1GPL	A	90	101
1GPL	A	285	296
1GPL	A	299	304
1GPQ	A	57	62
1GPQ	B	57	62
1GQV	A	62	71
1GW0	A	4	12
1GZ2	A	5	16
1GZ7	A	268	277
1H0J	A	42	53
1H0J	A	54	59
1H34	A	24	32
1H34	A	42	49
1H34	A	51	59
1H4I	B	6	12
1H4I	D	6	12

Additional file 1: Table S3: Disulphide loops at the surface of PDB proteins.

**PDB-ID**	**Chain-ID**	**Start**	**End**
1H6V	A	59	64
1H76	A	170	181
1H76	A	624	629
1H9H	E	128	136
1HA0	A	473	477
1HAQ	A	62	67
1HAQ	A	123	128
1HAQ	A	187	192
1HAQ	A	233	244
1HAQ	A	244	249
1HAQ	A	291	302
1HAQ	A	302	307
1HAQ	A	356	367
1HAQ	A	367	371
1HAQ	A	413	424
1HAQ	A	424	430
1HAQ	A	476	487
1HAQ	A	487	491
1HAQ	A	535	546

Additional file 1: Table S3: Disulphide loops at the surface of PDB proteins.

PDB-ID	Chain-ID	Start	End
1HAQ	A	546	551
1HAQ	A	605	612
1HAQ	A	655	666
1HAQ	A	666	673
1HAQ	A	715	726
1HAQ	A	726	735
1HAQ	A	763	774
1HAQ	A	774	785
1HAQ	A	785	793
1HAQ	A	835	846
1HAQ	A	846	852
1HAQ	A	897	908
1HAQ	A	908	913
1HAQ	A	955	966
1HAQ	A	966	971
1HAQ	A	1014	1025
1HAQ	A	1025	1030
1HAQ	A	1073	1084
1HAQ	A	1084	1091

Additional file 1: Table S3: Disulphide loops at the surface of PDB proteins.

**PDB-ID**	**Chain-ID**	**Start**	**End**
1HAQ	A	1134	1145
1HAQ	A	1145	1149
1HAQ	A	1200	1210
1HC1	A	93	98
1HC1	B	93	98
1HC1	C	93	98
1HC1	D	93	98
1HC1	E	93	98
1HC1	F	93	98
1HC9	A	29	33
1HC9	A	48	59
1HC9	A	60	65
1HCN	B	93	100
1HDM	B	25	35
1HG8	A	340	345
1HG8	A	364	371
1HGD	B	144	148
1HGD	D	144	148
1HGD	F	144	148

Additional file 1: Table S3: Disulphide loops at the surface of PDB proteins.

PDB-ID	Chain-ID	Start	End
1HIA	I	6	17
1HIA	I	11	22
1HKF	A	37	45
1HLG	A	227	236
1HLG	B	227	236
1HM4	A	128	136
1HMC	A	139	146
1HMC	B	139	146
1HPB	P	38	45
1HPL	A	4	10
1HPL	A	90	101
1HPL	A	285	296
1HPL	A	299	304
1HQ8	A	112	121
1HQ8	A	115	126
1HSL	A	38	45
1HSL	B	38	45
1HTN	A	50	60
1HVQ	A	50	57

Additional file 1: Table S3: Disulphide loops at the surface of PDB proteins.

PDB-ID	Chain-ID	Start	End
1HYR	A	96	105
1HYR	A	99	110
1HYR	B	96	105
1HYR	B	99	110
1HYR	C	36	41
1I1R	A	112	122
1I1R	A	150	160
1I1R	B	31	37
1I1R	B	60	70
1I8N	A	75	84
1I9W	A	62	68
1I9W	A	63	68
1I9W	A	68	78
1I9W	A	301	306
1I9W	A	370	376
1I9W	A	370	380
1I9W	A	376	380
1IA5	A	306	311
1IA5	A	330	339

Additional file 1: Table S3: Disulphide loops at the surface of PDB proteins.

**PDB-ID**	**Chain-ID**	**Start**	**End**
1IAG	A	157	164
1IAR	B	9	19
1ICF	I	227	234
1IDJ	A	302	310
1IGA	A	199	207
1IGA	A	304	314
1IGA	B	199	207
1IGA	B	304	314
1IGR	A	185	194
1IGR	A	189	200
1IGR	A	201	209
1IGR	A	221	230
1IGR	A	294	298
1IHP	A	8	17
1IHP	A	413	421
1IIU	A	120	129
1IJL	A	75	86
1IJL	B	75	86
1IKP	A	11	15

Additional file 1: Table S3: Disulphide loops at the surface of PDB proteins.

**PDB-ID**	**Chain-ID**	**Start**	**End**
1IKP	A	372	379
1ILL	G	6	16
1ILL	R	4	14
1ILM	B	10	20
1ILM	G	6	16
1ING	A	124	129
1ING	A	232	237
1ING	A	280	289
1ING	B	124	129
1ING	B	232	237
1ING	B	280	289
1IOD	A	2	13
1IOD	B	202	213
1IOD	G	417	422
1IOO	A	16	21
1IOO	A	169	180
1IQ9	A	42	53
1IQ9	A	54	59
1IQQ	A	15	22

Additional file 1: Table S3: Disulphide loops at the surface of PDB proteins.

PDB-ID	Chain-ID	Start	End
1IQQ	A	170	181
1ISF	A	239	248
1ISF	B	239	248
1ITO	A	63	67
1ITO	A	108	119
1IVO	A	166	175
1IVO	A	191	199
1IVO	A	208	216
1IVO	A	227	236
1IVO	A	305	309
1IVO	A	482	491
1IVO	A	502	511
1IVO	B	166	175
1IVO	B	191	199
1IVO	B	208	216
1IVO	B	227	236
1IVO	B	305	309
1IVO	B	482	491
1IVO	B	502	511

Additional file 1: Table S3: Disulphide loops at the surface of PDB proteins.

PDB-ID	Chain-ID	Start	End
1IVO	C	33	42
1IVO	D	33	42
1IVY	A	213	218
1IVY	B	213	218
1IXX	A	2	13
1IXX	B	2	13
1IXX	C	2	13
1IXX	D	2	13
1IXX	E	2	13
1IXX	F	2	13
1J2E	A	328	339
1J2E	A	385	394
1J2E	B	328	339
1J2E	B	385	394
1J2L	A	6	14
1J2L	A	27	33
1J34	A	2	13
1J34	B	202	213
1J34	C	418	423

Additional file 1: Table S3: Disulphide loops at the surface of PDB proteins.

PDB-ID	Chain-ID	Start	End
1J36	A	133	141
1J78	A	95	106
1J78	A	173	182
1J78	A	249	257
1J78	A	359	368
1J78	A	436	446
1J8J	A	47	52
1JBL	A	3	11
1JEH	A	44	49
1JEH	B	44	49
1JHN	A	361	367
1JK4	A	28	34
1JK4	A	74	79
1JK4	B	1	6
1JMA	A	118	127
1JMA	B	4	15
1JMA	B	89	97
1JNF	A	171	177
1JNF	A	494	503

Additional file 1: Table S3: Disulphide loops at the surface of PDB proteins.

PDB-ID	Chain-ID	Start	End
1JNF	A	612	617
1JPE	A	103	109
1JS8	A	2549	2559
1JS8	A	2815	2821
1JV2	A	59	67
1JV2	A	461	472
1JV2	A	596	602
1JV2	A	874	879
1JV2	B	177	184
1JV2	B	536	544
1JV2	B	542	547
1JV2	B	549	558
1JV2	B	575	586
1JV2	B	588	598
1JW1	A	170	181
1JW1	A	625	630
1JWI	A	4	15
1JWI	B	4	15
1JX3	A	169	176

Additional file 1: Table S3: Disulphide loops at the surface of PDB proteins.

**PDB-ID**	**Chain-ID**	**Start**	**End**
1JX5	A	56	65
1JZN	A	3	14
1JZN	B	3	14
1JZN	C	3	14
1JZN	D	3	14
1JZN	E	3	14
1K4Y	A	273	284
1K7T	A	35	40
1K7T	A	78	83
1K7T	A	121	126
1K7T	A	164	169
1K7T	B	35	40
1K7T	B	78	83
1K7T	B	121	126
1K7T	B	164	169
1K8I	B	24	34
1K8Q	A	227	236
1K9I	A	256	267
1K9I	B	256	267

Additional file 1: Table S3: Disulphide loops at the surface of PDB proteins.

PDB-ID	Chain-ID	Start	End
1K9I	C	256	267
1K9I	D	256	267
1K9I	E	256	267
1K9I	F	256	267
1K9I	G	256	267
1K9I	H	256	267
1K9I	I	256	267
1K9I	J	256	267
1K9J	A	268	279
1K9J	B	268	279
1KBA	A	27	31
1KBA	A	59	64
1KBA	B	27	31
1KBA	B	59	64
1KCG	A	96	105
1KCG	A	99	110
1KCG	B	96	105
1KCG	B	99	110
1KGY	A	105	115

Additional file 1: Table S3: Disulphide loops at the surface of PDB proteins.

PDB-ID	Chain-ID	Start	End
1KGY	D	705	715
1KIG	H	22	27
1KIG	L	389	400
1KLA	A	7	16
1KLA	B	7	16
1KLO	A	11	19
1KLO	A	33	42
1KLO	A	90	99
1KLO	A	145	154
1KLX	A	22	30
1KLX	A	52	60
1KLX	A	88	96
1KLX	A	124	132
1KP6	A	5	12
1KTB	A	25	32
1KTZ	B	38	44
1KTZ	B	115	120
1KUR	A	50	60
1KUR	A	65	71

Additional file 1: Table S3: Disulphide loops at the surface of PDB proteins.

PDB-ID	Chain-ID	Start	End
1KUR	A	126	136
1KUR	A	140	149
1KUR	A	150	158
1KWI	A	85	96
1KWN	A	56	66
1KWN	A	71	77
1KWN	A	134	145
1KWN	A	149	158
1KWN	A	159	164
1L9L	A	34	45
1LAF	E	38	45
1LBE	A	239	248
1LBE	B	239	248
1LCG	A	109	114
1LCG	A	272	276
1LCR	A	170	175
1LCS	A	133	139
1LGY	A	235	244
1LH9	A	127	134

Additional file 1: Table S3: Disulphide loops at the surface of PDB proteins.

PDB-ID	Chain-ID	Start	End
1LH9	A	397	406
1LH9	A	411	420
1LH9	A	441	452
1LHA	A	408	417
1LHA	A	442	453
1LI1	A	65	71
1LI1	A	176	182
1LI1	B	65	71
1LI1	B	176	182
1LI1	C	65	71
1LI1	C	176	182
1LI1	D	65	71
1LI1	D	176	182
1LI1	E	65	71
1LI1	E	176	182
1LI1	F	65	71
1LI1	F	176	182
1LIT	A	14	25
1LK1	C	29	33

Additional file 1: Table S3: Disulphide loops at the surface of PDB proteins.

**PDB-ID**	**Chain-ID**	**Start**	**End**
1LK1	C	48	59
1LK1	C	60	65
1LK1	F	29	33
1LK1	F	48	59
1LK1	F	60	65
1LK9	A	41	50
1LK9	A	368	376
1LK9	B	41	50
1LK9	B	368	376
1LMG	A	46	57
1LMG	A	58	63
1LN7	A	44	54
1LN7	A	55	60
1LN9	A	46	57
1LN9	A	58	63
1LNK	A	42	53
1LNK	A	54	59
1LNL	A	47	58
1LNL	A	328	334

Additional file 1: Table S3: Disulphide loops at the surface of PDB proteins.

PDB-ID	Chain-ID	Start	End
1LNL	B	47	58
1LNL	B	328	334
1LNL	C	47	58
1LNL	C	328	334
1LPF	A	48	53
1LPF	B	48	53
1LQ3	A	46	57
1LQ3	A	58	63
1LQV	C	17	22
1LR7	A	66	77
1LRW	B	6	12
1LRW	D	6	12
1LVL	A	43	48
1LX1	A	79	90
1LYA	A	46	53
1LYA	B	222	226
1LYA	C	46	53
1LYA	D	222	226
1M11	2	112	121

Additional file 1: Table S3: Disulphide loops at the surface of PDB proteins.

PDB-ID	Chain-ID	Start	End
1M11	2	121	126
1M11	2	126	134
1M11	3	208	212
1M11	3	208	217
1M11	3	212	217
1M11	R	60	64
1M11	R	124	129
1M11	R	186	191
1M1V	A	159	164
1M3D	A	65	71
1M3D	A	176	182
1M3D	B	65	71
1M3D	B	176	182
1M3D	C	64	70
1M3D	C	173	180
1M3D	D	65	71
1M3D	D	176	182
1M3D	E	65	71
1M3D	E	176	182

Additional file 1: Table S3: Disulphide loops at the surface of PDB proteins.

**PDB-ID**	**Chain-ID**	**Start**	**End**
1M3D	F	64	70
1M3D	F	173	180
1M4U	A	207	215
1M6B	A	167	175
1M6B	A	191	199
1M6B	A	208	216
1M6B	A	227	236
1M6B	A	271	282
1M6B	A	304	308
1M6B	A	481	490
1M6B	A	501	510
1M6B	A	557	566
1M92	A	113	120
1MCT	I	16	27
1MF3	A	107	118
1MGU	A	107	118
1MHL	C	115	125
1MHL	C	221	232
1MHL	D	115	125

Additional file 1: Table S3: Disulphide loops at the surface of PDB proteins.

**PDB-ID**	**Chain-ID**	**Start**	**End**
1MHL	D	221	232
1MN1	A	341	348
1MNW	A	62	66
1MO9	A	82	87
1MO9	B	82	87
1MOF	A	86	93
1MOX	A	166	175
1MOX	A	191	199
1MOX	A	208	216
1MOX	A	227	236
1MOX	A	305	309
1MOX	A	482	491
1MOX	B	166	175
1MOX	B	191	199
1MOX	B	208	216
1MOX	B	227	236
1MOX	B	305	309
1MOX	B	482	491
1MOX	C	34	43

Additional file 1: Table S3: Disulphide loops at the surface of PDB proteins.

PDB-ID	Chain-ID	Start	End
1MOX	D	34	43
1MPP	A	45	50
1MQL	B	144	148
1MQL	E	144	148
1MQL	H	144	148
1MWP	A	98	105
1MX1	A	1274	1285
1MX1	B	2274	2285
1MX1	C	3274	3285
1MX1	D	4274	4285
1MX1	E	5274	5285
1MX1	F	6274	6285
1N0L	A	207	212
1N10	A	1078	1084
1N26	A	102	113
1N26	A	146	157
1N4Y	A	6	14
1N4Y	A	27	33
1N69	A	36	47

Additional file 1: Table S3: Disulphide loops at the surface of PDB proteins.

PDB-ID	Chain-ID	Start	End
1N69	B	36	47
1N6G	A	105	116
1N6G	A	116	121
1N6G	B	105	116
1N6G	B	116	121
1N6G	C	105	116
1N6G	C	116	121
1N7D	A	297	308
1N7D	A	337	347
1N7Z	A	142	153
1N7Z	B	142	153
1N8Y	C	174	183
1N8Y	C	199	206
1N8Y	C	203	214
1N8Y	C	215	223
1N8Y	C	234	243
1N8Y	C	313	317
1N8Y	C	490	499
1N8Y	C	510	519

Additional file 1: Table S3: Disulphide loops at the surface of PDB proteins.

**PDB-ID**	**Chain-ID**	**Start**	**End**
1N8Y	C	566	575
1N8Z	C	173	182
1N8Z	C	198	205
1N8Z	C	202	213
1N8Z	C	214	222
1N8Z	C	233	242
1N8Z	C	312	316
1N8Z	C	489	498
1N8Z	C	509	518
1N8Z	C	565	574
1NCD	N	124	129
1NCD	N	232	237
1NCD	N	280	289
1ND1	A	159	164
1NEP	A	23	28
1NEP	A	74	80
1NHC	A	335	340
1NHC	A	359	368
1NJR	A	128	136

Additional file 1: Table S3: Disulphide loops at the surface of PDB proteins.

PDB-ID	Chain-ID	Start	End
1NL1	A	18	23
1NOT	A	2	7
1NOT	A	3	13
1NPE	A	1094	1105
1NPE	B	738	746
1NPE	B	760	769
1NPE	B	817	826
1NPE	B	872	881
1NST	A	818	828
1NTJ	A	61	66
1NTJ	A	123	128
1NTJ	A	194	200
1NTJ	A	255	261
1NTL	A	59	64
1NTL	A	121	126
1NTL	A	192	198
1NTL	A	254	260
1NTL	A	329	334
1NTL	A	329	336

Additional file 1: Table S3: Disulphide loops at the surface of PDB proteins.

**PDB-ID**	**Chain-ID**	**Start**	**End**
1NTL	A	331	336
1NTL	B	59	64
1NTL	B	121	126
1NTL	B	192	198
1NTL	B	254	260
1NTL	B	329	334
1NTL	B	329	336
1NTL	B	331	336
1NTN	A	27	31
1NTN	A	46	57
1NTN	A	58	63
1NYS	A	104	109
1NYS	B	4	12
1NYS	C	104	109
1O7D	A	268	273
1O7D	C	493	501
1O86	A	152	158
1OB1	C	7	18
1OB1	C	30	41

Additional file 1: Table S3: Disulphide loops at the surface of PDB proteins.

PDB-ID	Chain-ID	Start	End
1OBR	A	314	323
1OC0	B	25	31
1OGQ	A	34	43
1OGQ	A	305	312
1OGS	A	18	23
1OHT	A	50	56
1OLZ	A	76	87
1OLZ	A	105	114
1OLZ	B	76	87
1OLZ	B	105	114
1ONF	A	39	44
1ONJ	A	42	53
1ONJ	A	54	59
1OO1	A	78	89
1OQE	K	9	20
1OQE	L	9	20
1OQE	M	9	20
1OQE	N	9	20
1OQE	O	9	20

Additional file 1: Table S3: Disulphide loops at the surface of PDB proteins.

PDB-ID	Chain-ID	Start	End
1OQE	P	9	20
1OQE	Q	9	20
1OQE	R	9	20
1ORL	A	16	26
1ORV	A	385	394
1ORV	B	385	394
1ORV	C	385	394
1ORV	D	385	394
1OUV	A	56	64
1OUV	A	92	100
1OUV	A	128	136
1OUV	A	164	172
1OUV	A	200	208
1OUV	A	236	244
1OUV	A	272	280
1OVW	B	18	24
1OVW	B	60	66
1OVW	B	223	228
1OZ7	A	2	13

Additional file 1: Table S3: Disulphide loops at the surface of PDB proteins.

PDB-ID	Chain-ID	Start	End
1OZ7	B	2	13
1OZN	A	27	33
1P3Z	A	174	182
1P49	A	141	148
1P49	A	481	487
1P49	A	562	570
1P49	A	563	572
1P4L	D	145	150
1P58	A	105	116
1P58	A	116	121
1P58	B	105	116
1P58	B	116	121
1P58	C	105	116
1P58	C	116	121
1P9G	A	35	39
1PA2	A	44	49
1PB2	A	18	29
1PBI	A	14	22
1PBI	A	32	39

Additional file 1: Table S3: Disulphide loops at the surface of PDB proteins.

PDB-ID	Chain-ID	Start	End
1PBI	A	41	49
1PBI	B	14	22
1PBI	B	32	39
1PBI	B	41	49
1PCV	A	51	61
1PCV	A	66	72
1PCV	A	133	143
1PCV	A	147	156
1PCV	A	157	162
1PEN	A	2	8
1PFX	L	18	23
1PFX	L	51	62
1PFX	L	73	82
1PFX	L	88	99
1PGS	A	51	56
1PGS	A	204	208
1PI2	A	13	21
1PI2	A	30	37
1PI2	A	39	47

Additional file 1: Table S3: Disulphide loops at the surface of PDB proteins.

PDB-ID	Chain-ID	Start	End
1PJA	A	109	117
1PJA	A	165	176
1POC	A	105	113
1PRE	A	159	164
1PRE	B	159	164
1PU4	A	734	741
1PU4	B	734	741
1PVH	A	112	122
1PVH	A	150	160
1PXZ	A	285	291
1Q47	A	103	114
1Q47	A	132	141
1Q47	B	103	114
1Q8D	A	250	256
1Q8D	A	315	325
1Q8H	A	23	29
1QCX	A	303	311
1QDM	A	49	55
1QDM	A	214	218

Additional file 1: Table S3: Disulphide loops at the surface of PDB proteins.

PDB-ID	Chain-ID	Start	End
1QFX	A	394	402
1QFX	B	394	402
1QHU	A	126	131
1QI9	A	77	86
1QI9	A	544	555
1QI9	B	77	86
1QI9	B	544	555
1QKD	A	43	54
1QKD	A	55	60
1QKD	B	43	54
1QKD	B	55	60
1QLL	A	75	86
1QLL	B	75	86
1QO3	C	145	150
1QO3	D	145	150
1QWO	A	8	17
1QWO	A	413	421
1R2M	A	53	64
1R42	A	133	141

Additional file 1: Table S3: Disulphide loops at the surface of PDB proteins.

PDB-ID	Chain-ID	Start	End
1R46	A	56	63
1R46	A	378	382
1R46	B	56	63
1R46	B	378	382
1R54	A	361	371
1R8N	A	139	147
1R9L	A	136	142
1RC9	A	167	174
1RC9	A	170	179
1RD8	B	144	148
1RD8	D	144	148
1RD8	F	144	148
1RDS	A	3	11
1RFN	B	88	99
1RLX	A	6	11
1RMG	A	322	328
1RMG	A	350	359
1RMR	A	20	26
1RN7	A	75	85

Additional file 1: Table S3: Disulphide loops at the surface of PDB proteins.

PDB-ID	Chain-ID	Start	End
1RNF	A	64	71
1RP1	A	4	10
1RP1	A	90	101
1RP1	A	285	296
1RP1	A	299	304
1RP4	A	100	105
1RPA	A	315	319
1RRA	A	65	72
1RUY	I	644	648
1RUY	K	644	648
1RUY	M	644	648
1S2B	A	141	148
1S2B	A	194	203
1S2J	A	48	54
1S4Y	A	82	87
1S4Y	B	4	12
1S4Y	C	82	87
1S4Y	D	4	12
1S78	A	173	182

Additional file 1: Table S3: Disulphide loops at the surface of PDB proteins.

PDB-ID	Chain-ID	Start	End
1S78	A	198	205
1S78	A	202	213
1S78	A	214	222
1S78	A	233	242
1S78	A	312	316
1S78	A	489	498
1S78	A	509	518
1S8M	A	410	417
1SB2	A	2	13
1SB2	B	4	15
1SCH	B	44	49
1SFR	A	87	92
1SG1	X	4	15
1SHW	B	305	315
1SHY	B	95	101
1SHY	B	133	141
1SHY	B	541	551
1SK3	A	214	220
1SKZ	A	8	19

Additional file 1: Table S3: Disulphide loops at the surface of PDB proteins.

PDB-ID	Chain-ID	Start	End
1SKZ	A	62	73
1SMR	A	45	50
1SMR	A	206	210
1T0P	A	287	294
1T2Z	A	95	105
1T7H	A	5	13
1T7H	B	5	13
1TA3	B	254	260
1TAB	I	24	32
1TBI	A	68	73
1TEJ	A	20	26
1TEJ	B	20	26
1TFG	A	7	16
1TGL	A	29	40
1TGL	A	235	244
1TIA	A	36	41
1TIC	A	29	40
1TIC	A	235	244
1TIE	A	132	139

Additional file 1: Table S3: Disulphide loops at the surface of PDB proteins.

PDB-ID	Chain-ID	Start	End
1TZP	A	216	223
1TZP	B	216	223
1U0O	A	2	13
1U0O	B	202	213
1U79	A	106	111
1UG4	A	42	53
1UG4	A	54	59
1UHA	A	36	40
1UHA	A	77	81
1UKM	A	4	15
1UKM	B	1	12
1ULK	A	36	40
1ULK	A	77	81
1ULK	A	118	122
1ULK	B	236	240
1ULK	B	277	281
1ULK	B	318	322
1UMR	A	4	15
1UMR	C	204	215

Additional file 1: Table S3: Disulphide loops at the surface of PDB proteins.

PDB-ID	Chain-ID	Start	End
1USW	A	227	234
1UUZ	A	59	64
1UV0	A	14	25
1UV9	A	473	484
1UV9	A	602	608
1UZJ	A	1491	1502
1V0X	A	21	32
1V0X	A	44	54
1V0X	A	555	561
1V0Z	A	130	135
1V0Z	A	239	244
1V0Z	A	287	296
1V0Z	B	130	135
1V0Z	B	239	244
1V0Z	B	287	296
1V0Z	C	130	135
1V0Z	C	239	244
1V0Z	C	287	296
1V0Z	D	130	135

Additional file 1: Table S3: Disulphide loops at the surface of PDB proteins.

PDB-ID	Chain-ID	Start	End
1V0Z	D	239	244
1V0Z	D	287	296
1V4L	A	4	15
1V4L	B	204	215
1V4L	C	4	15
1V4L	D	204	215
1V4L	E	4	15
1V4L	F	204	215
1VAP	A	75	86
1VKJ	A	260	269
1VKJ	B	260	269
1VKJ	C	260	269
1VYE	A	23	33
1VYE	A	26	33
1VYE	A	33	39
1VYE	A	39	46
1VYE	A	64	68
1VZM	A	17	23
1W0R	A	5	16

Additional file 1: Table S3: Disulphide loops at the surface of PDB proteins.

**PDB-ID**	**Chain-ID**	**Start**	**End**
1W0R	A	62	66
1W0R	A	66	77
1W0R	A	77	84
1W0R	A	100	105
1W0R	A	100	106
1W0R	A	121	125
1W0R	A	125	136
1W0R	A	136	143
1W0R	A	136	147
1W0R	A	143	147
1W0R	A	147	157
1W0R	A	157	163
1W0R	A	178	182
1W0R	A	211	221
1W0R	A	221	227
1W0R	A	242	246
1W0R	A	246	257
1W0R	A	269	279
1W0R	A	279	285

Additional file 1: Table S3: Disulphide loops at the surface of PDB proteins.

PDB-ID	Chain-ID	Start	End
1W0R	A	300	310
1W0R	A	323	333
1W0R	A	333	343
1W0R	A	343	349
1W0R	A	364	368
1W0R	A	428	434
1W1C	A	59	64
1W1C	B	59	64
1W1E	A	61	66
1W1E	B	61	66
1W8A	A	543	549
1W8A	A	547	556
1WBA	A	135	141
1WC2	A	103	113
1WD3	A	21	31
1WD3	A	81	86
1WGC	A	35	40
1WGC	A	78	83
1WGC	A	121	126

Additional file 1: Table S3: Disulphide loops at the surface of PDB proteins.

PDB-ID	Chain-ID	Start	End
1WGC	A	164	169
1WQJ	I	47	52
1WUW	A	16	25
1WUW	B	66	75
1WVR	A	167	174
1WVR	A	170	179
1WWL	A	8	17
1WWL	B	8	17
1X8Z	A	8	17
1XCD	A	25	31
1XCD	A	29	38
1XDT	R	134	143
1XDT	T	461	471
1XED	A	38	46
1XFD	A	411	418
1XFD	B	411	418
1XG2	B	9	18
1XTA	A	167	174
1XTA	A	170	179

Additional file 1: Table S3: Disulphide loops at the surface of PDB proteins.

PDB-ID	Chain-ID	Start	End
1XTA	B	167	174
1XTA	B	170	179
1XU1	R	89	100
1XU1	R	93	104
1XU1	S	89	100
1XU1	S	93	104
1XU1	T	89	100
1XU1	T	93	104
1XWD	B	87	94
1XWD	C	18	25
1XWD	C	23	32
1XX1	A	51	57
1XX1	B	51	57
1Y17	A	2	13
1Y17	B	2	13
1Y1E	X	336	341
1Y4M	A	86	93
1Y4M	B	86	93
1Y4M	C	86	93

Additional file 1: Table S3: Disulphide loops at the surface of PDB proteins.

PDB-ID	Chain-ID	Start	End
1Y68	A	46	57
1Y68	A	58	63
1YCK	A	46	52
1YCV	A	38	49
1YCV	A	50	55
1YG9	A	45	50
1YG9	A	237	245
1YO8	A	553	564
1YO8	A	577	588
1YO8	A	680	691
1YO8	A	707	715
1YPO	A	144	155
1YPO	B	144	155
1YPQ	A	144	155
1YPQ	B	144	155
1YUK	B	437	448
1YUK	B	450	459
1YWH	A	6	12
1YWH	A	71	76

Additional file 1: Table S3: Disulphide loops at the surface of PDB proteins.

**PDB-ID**	**Chain-ID**	**Start**	**End**
1YWH	A	98	105
1YWH	A	171	176
1YWH	A	197	205
1YWH	A	266	271
1YWH	C	6	12
1YWH	C	71	76
1YWH	C	98	105
1YWH	C	171	176
1YWH	C	197	205
1YWH	C	266	271
1YWH	E	6	12
1YWH	E	71	76
1YWH	E	98	105
1YWH	E	171	176
1YWH	E	197	205
1YWH	E	266	271
1YWH	G	6	12
1YWH	G	71	76
1YWH	G	98	105

Additional file 1: Table S3: Disulphide loops at the surface of PDB proteins.

**PDB-ID**	**Chain-ID**	**Start**	**End**
1YWH	G	171	176
1YWH	G	197	205
1YWH	G	266	271
1YWH	I	6	12
1YWH	I	71	76
1YWH	I	98	105
1YWH	I	171	176
1YWH	I	197	205
1YWH	I	266	271
1YWH	K	6	12
1YWH	K	71	76
1YWH	K	98	105
1YWH	K	171	176
1YWH	K	197	205
1YWH	K	266	271
1YWH	M	6	12
1YWH	M	71	76
1YWH	M	98	105
1YWH	M	171	176

Additional file 1: Table S3: Disulphide loops at the surface of PDB proteins.

PDB-ID	Chain-ID	Start	End
1YWH	M	197	205
1YWH	M	266	271
1YWH	O	6	12
1YWH	O	71	76
1YWH	O	98	105
1YWH	O	171	176
1YWH	O	197	205
1YWH	O	266	271
1Z1X	A	20	26
1Z4V	A	365	375
1Z4V	A	448	458
1Z4V	A	528	539
1Z68	A	321	332
1Z68	B	321	332
1Z6I	A	390	396
1Z7C	A	182	189
1Z8Y	A	68	78
1Z8Y	C	68	78
1Z8Y	E	68	78

Additional file 1: Table S3: Disulphide loops at the surface of PDB proteins.

PDB-ID	Chain-ID	Start	End
1Z8Y	G	68	78
1ZEI	A	38	43
1ZEI	B	38	43
1ZEI	C	38	43
1ZEI	D	38	43
1ZEI	E	38	43
1ZEI	F	38	43
1ZJS	A	21	29
1ZJV	A	11	22
1ZK7	A	42	47
1ZMC	A	45	50
1ZMC	B	45	50
1ZOX	A	36	44
1ZRH	A	223	232
1ZT3	A	217	228
1ZTM	A	331	340
1ZTM	A	355	363
1ZTM	A	387	392
1ZTM	B	331	340

Additional file 1: Table S3: Disulphide loops at the surface of PDB proteins.

PDB-ID	Chain-ID	Start	End
1ZTM	B	355	363
1ZTM	B	387	392
1ZTM	C	331	340
1ZTM	C	355	363
1ZTM	C	387	392
2A73	B	1484	1489
2A73	B	1615	1624
2A8X	A	41	46
2A8X	B	41	46
2AAA	A	30	38
2AGC	A	68	75
2AGC	A	94	105
2AHN	A	57	67
2AHN	A	72	79
2AHN	A	160	169
2AHN	A	170	180
2AHX	A	164	172
2AHX	A	188	196
2AHX	A	205	213

Additional file 1: Table S3: Disulphide loops at the surface of PDB proteins.

PDB-ID	Chain-ID	Start	End
2AHX	A	224	233
2AHX	A	268	279
2AHX	A	301	305
2AHX	A	478	487
2AHX	A	498	507
2AHX	A	555	564
2AHX	A	592	600
2AO7	A	503	511
2AOZ	A	75	86
2ARP	A	4	12
2ARP	F	66	77
2ARP	F	139	150
2ASI	A	51	57
2ATY	A	63	72
2ATY	A	154	159
2ATY	A	154	161
2ATY	A	156	161
2ATY	B	63	72
2ATY	B	154	159

Additional file 1: Table S3: Disulphide loops at the surface of PDB proteins.

PDB-ID	Chain-ID	Start	End
2ATY	B	154	161
2ATY	B	156	161
2AW2	A	72	79
2AW2	B	4	15
2AW2	B	89	97
2B0U	A	4	12
2B0U	B	4	12
2B0U	C	66	77
2B0U	C	139	150
2B0U	C	216	227
2B0U	D	66	77
2B0U	D	139	150
2B0U	D	216	227
2B39	A	1504	1509
2B39	A	1635	1644
2B5E	A	90	97
2B9B	A	324	333
2B9B	A	348	356
2B9B	A	380	385

Additional file 1: Table S3: Disulphide loops at the surface of PDB proteins.

PDB-ID	Chain-ID	Start	End
2B9B	B	324	333
2B9B	B	348	356
2B9B	B	380	385
2B9B	C	324	333
2B9B	C	348	356
2B9B	C	380	385
2BBA	A	97	107
2BO2	A	5	15
2BPD	A	119	130
2BPD	B	119	130
2C6F	A	128	136
2C6J	A	29	36
2C6U	A	102	113
2C9A	A	27	36
2CAS	A	490	494
2CH9	A	99	110
2CKV	Y	365	370
2CMZ	A	153	158
2CMZ	B	153	158

Additional file 1: Table S3: Disulphide loops at the surface of PDB proteins.

PDB-ID	Chain-ID	Start	End
2CMZ	C	153	158
2COV	D	413	418
2COV	E	413	418
2COV	F	413	418
2COV	G	413	418
2COV	H	413	418
2COV	I	413	418
2CTX	A	26	30
2CTX	A	45	56
2CTX	A	57	62
2D9Q	A	36	42
2D9Q	A	64	74
2D9Q	B	108	119
2D9Q	B	154	163
2DDA	A	157	164
2DDA	A	160	169
2DDB	A	156	163
2DDB	A	159	168
2DDU	A	1339	1348

Additional file 1: Table S3: Disulphide loops at the surface of PDB proteins.

**PDB-ID**	**Chain-ID**	**Start**	**End**
2DDU	A	1413	1423
2DDU	A	1417	1428
2DDU	A	1430	1441
2DKV	A	67	71
2DKV	A	184	192
2DRF	B	279	289
2DSQ	G	192	203
2DSQ	I	47	52
2DTG	E	192	201
2DTG	E	196	207
2DTG	E	208	216
2DTG	E	228	237
2DTG	E	266	274
2DTG	E	304	308
2DTG	E	786	795
2DW0	A	350	355
2DW0	A	417	423
2DW0	A	441	447
2DW0	A	538	548

Additional file 1: Table S3: Disulphide loops at the surface of PDB proteins.

**PDB-ID**	**Chain-ID**	**Start**	**End**
2DW0	A	591	602
2DYP	D	133	143
2DYZ	A	24	34
2DYZ	A	34	39
2DYZ	A	83	88
2DYZ	A	109	117
2E26	A	2059	2070
2E26	A	2133	2143
2E26	A	2151	2160
2E26	A	2482	2492
2E26	A	2486	2497
2E26	A	2499	2508
2E3X	A	162	167
2E3X	A	229	235
2E3X	A	253	259
2E3X	A	350	360
2E3X	B	2	13
2E3X	C	4	15
2E4U	A	412	419

Additional file 1: Table S3: Disulphide loops at the surface of PDB proteins.

PDB-ID	Chain-ID	Start	End
2E4U	B	412	419
2E4Z	A	430	437
2E9W	A	443	450
2E9W	B	443	450
2EAV	A	246	252
2EAV	B	246	252
2EBO	A	601	608
2EBO	B	601	608
2EBO	C	601	608
2ERO	A	352	357
2ERO	A	419	425
2ERO	A	443	449
2ERO	A	539	549
2ERO	A	592	603
2ERO	B	352	357
2ERO	B	419	425
2ERO	B	443	449
2ERO	B	539	549
2ERO	B	592	603

Additional file 1: Table S3: Disulphide loops at the surface of PDB proteins.

**PDB-ID**	**Chain-ID**	**Start**	**End**
2F2L	A	390	396
2F2L	X	371	377
2F83	A	32	38
2F83	A	122	128
2F83	A	212	218
2F83	A	303	309
2FHU	B	144	148
2FHU	D	144	148
2FHU	F	144	148
2FHV	B	144	148
2FHV	D	144	148
2FHV	F	144	148
2FMW	A	29	38
2FRG	P	52	59
2FT3	A	27	33
2FT3	A	31	40
2FT3	B	27	33
2FT3	B	31	40
2FZ6	A	58	69

Additional file 1: Table S3: Disulphide loops at the surface of PDB proteins.

PDB-ID	Chain-ID	Start	End
2G3P	A	46	53
2G3P	B	46	53
2G81	I	24	32
2G81	I	42	49
2G81	I	51	59
2GAK	A	372	381
2GAK	B	372	381
2GBC	A	326	337
2GBC	B	326	337
2GHN	A	77	88
2GIY	A	280	289
2GIY	A	314	323
2GIY	B	280	289
2GIY	B	314	323
2GJ1	A	115	125
2GJ1	A	221	232
2GP2	A	154	159
2GSX	A	62	71
2GSX	A	126	134

Additional file 1: Table S3: Disulphide loops at the surface of PDB proteins.

PDB-ID	Chain-ID	Start	End
2GSX	A	190	195
2GSX	A	251	256
2GSX	A	322	331
2GSX	A	386	390
2GSX	A	446	451
2GSX	A	502	507
2GSX	A	573	582
2GSX	A	637	642
2GSX	A	694	699
2GSX	A	759	768
2GSX	A	823	831
2GSX	A	887	892
2GUY	A	30	38
2GY5	A	211	220
2GY5	A	224	233
2GY5	A	242	251
2GY5	A	255	264
2GY5	A	268	274
2GY5	A	280	287

Additional file 1: Table S3: Disulphide loops at the surface of PDB proteins.

PDB-ID	Chain-ID	Start	End
2GY5	A	289	298
2GY5	A	302	311
2GY5	A	315	323
2GY5	A	331	340
2H5F	A	13	21
2H5F	A	57	68
2H5F	A	69	74
2H62	C	40	44
2H62	C	102	107
2H62	D	86	91
2H7Z	A	13	21
2H7Z	A	55	66
2H7Z	A	67	72
2H7Z	B	13	21
2H7Z	B	57	68
2H7Z	B	69	74
2H8I	A	75	86
2H8I	B	75	86
2HAU	A	171	177

Additional file 1: Table S3: Disulphide loops at the surface of PDB proteins.

PDB-ID	Chain-ID	Start	End
2HAU	A	495	506
2HAU	A	615	620
2HCZ	X	78	84
2HEV	R	31	42
2HLE	A	97	107
2HLQ	A	118	123
2HQM	A	61	66
2HQM	B	61	66
2HT5	A	124	129
2HT5	A	232	237
2HT5	A	280	289
2I0W	A	51	61
2I0W	A	66	72
2I0W	A	133	143
2I0W	A	147	156
2I0W	A	157	162
2ID5	A	3	9
2ID5	A	7	18
2ID5	B	3	9

Additional file 1: Table S3: Disulphide loops at the surface of PDB proteins.

PDB-ID	Chain-ID	Start	End
2ID5	B	7	18
2ID5	C	3	9
2ID5	C	7	18
2ID5	D	3	9
2ID5	D	7	18
2IDP	A	3	14
2IDP	B	3	14
2IFG	A	36	41
2IFG	A	40	50
2IFG	B	36	41
2IFG	B	40	50
2IJ6	A	3	14
2IJ6	B	3	14
2IL7	A	35	46
2ILO	A	3	14
2ILO	B	3	14
2IMV	A	2	13
2IMV	B	2	13
2IMX	A	3	14

Additional file 1: Table S3: Disulphide loops at the surface of PDB proteins.

PDB-ID	Chain-ID	Start	End
2IMX	B	3	14
2INM	A	3	14
2INM	B	3	14
2INO	A	3	14
2INO	B	3	14
2J04	B	375	383
2J2Z	A	207	212
2JA4	A	342	350
2JH1	A	37	45
2JH1	A	87	97
2JH1	A	177	187
2JIH	A	362	367
2JIH	A	534	545
2JQP	A	6	11
2JQP	A	46	57
2JQP	A	58	63
2JUQ	A	2	8
2JUQ	A	3	12
2JX9	A	96	102

Additional file 1: Table S3: Disulphide loops at the surface of PDB proteins.

PDB-ID	Chain-ID	Start	End
2KGU	A	10	21
2KGU	A	23	30
2KL5	A	88	92
2KUY	A	12	21
2KVD	A	40	51
2L03	A	7	14
2L03	A	67	72
2L1Q	A	17	28
2L1Q	A	23	33
2L3I	A	4	10
2L3Y	A	49	55
2L3Y	A	78	88
2L7S	A	16	21
2LB7	A	37	41
2LDF	A	3	11
2MCM	A	36	46
2MCM	A	88	93
2NMS	A	54	62
2NVK	X	57	62

Additional file 1: Table S3: Disulphide loops at the surface of PDB proteins.

PDB-ID	Chain-ID	Start	End
2NXZ	A	228	239
2OCW	A	38	46
2OCW	A	253	261
2OCW	A	367	377
2OCW	A	464	468
2OCW	A	468	478
2OCW	A	478	485
2OF6	A	105	116
2OF6	A	116	121
2OF6	B	105	116
2OF6	B	116	121
2OF6	C	105	116
2OF6	C	116	121
2OO4	A	1632	1639
2OR7	A	32	43
2OR8	A	31	42
2OTP	A	132	142
2OTP	B	132	142
2OX8	A	607	618

Additional file 1: Table S3: Disulphide loops at the surface of PDB proteins.

**PDB-ID**	**Chain-ID**	**Start**	**End**
2OX9	A	607	618
2OXE	A	21	27
2OXE	A	109	120
2OXE	A	304	315
2OXE	A	318	323
2OY3	A	487	497
2P26	A	437	448
2P26	A	450	459
2P26	A	484	495
2PE4	A	358	369
2PE4	A	420	429
2PLH	A	16	25
2PM8	A	252	263
2PM8	B	252	263
2PPL	A	21	27
2PPL	A	109	120
2PPL	A	303	314
2PPL	A	317	322
2Q5T	A	11	15

Additional file 1: Table S3: Disulphide loops at the surface of PDB proteins.

PDB-ID	Chain-ID	Start	End
2Q5T	A	394	401
2Q7N	A	10	20
2Q7N	A	37	45
2Q7N	A	293	303
2Q7Z	A	58	63
2Q7Z	A	120	125
2Q7Z	A	191	197
2Q7Z	A	252	256
2Q7Z	A	312	317
2Q7Z	A	375	380
2Q7Z	A	446	452
2Q7Z	A	508	513
2Q7Z	A	570	575
2Q7Z	A	641	647
2Q7Z	A	702	706
2Q7Z	A	762	767
2Q7Z	A	825	830
2Q7Z	A	896	902
2Q7Z	A	958	963

Additional file 1: Table S3: Disulphide loops at the surface of PDB proteins.

PDB-ID	Chain-ID	Start	End
2Q7Z	A	1020	1025
2Q7Z	A	1091	1097
2Q7Z	A	1152	1156
2Q7Z	A	1212	1217
2Q7Z	A	1275	1280
2Q7Z	A	1346	1355
2Q7Z	A	1411	1416
2Q7Z	A	1473	1478
2Q7Z	A	1544	1550
2Q7Z	A	1605	1609
2Q7Z	A	1665	1670
2Q7Z	A	1728	1733
2Q7Z	A	1799	1807
2Q7Z	A	1863	1868
2Q87	A	33	41
2Q87	B	33	41
2Q87	C	33	41
2QAE	A	41	46
2QAE	B	41	46

Additional file 1: Table S3: Disulphide loops at the surface of PDB proteins.

PDB-ID	Chain-ID	Start	End
2QBX	A	105	115
2QC1	A	29	33
2QC1	A	48	59
2QC1	A	60	65
2QJ4	A	75	85
2QJ4	B	75	85
2QLY	A	573	584
2QN4	A	140	144
2QN5	B	34	41
2QN5	B	81	89
2QN5	B	98	105
2QTS	A	173	180
2QTS	B	173	180
2QTS	C	173	180
2R0R	A	36	47
2R0R	B	36	47
2R2K	A	43	49
2R2K	B	43	49
2R2K	C	43	49

Additional file 1: Table S3: Disulphide loops at the surface of PDB proteins.

PDB-ID	Chain-ID	Start	End
2R2K	D	43	49
2R6P	A	105	116
2R6P	A	116	121
2R6P	B	105	116
2R6P	B	116	121
2R6P	C	105	116
2R6P	C	116	121
2RJP	A	322	327
2RJP	A	495	506
2RJQ	A	371	376
2RJQ	A	542	553
2RKQ	A	48	54
2RQZ	A	5	16
2RQZ	A	27	36
2UY6	A	207	212
2V5E	A	161	167
2V5E	A	216	221
2V5E	A	250	256
2V5E	A	315	325

Additional file 1: Table S3: Disulphide loops at the surface of PDB proteins.

PDB-ID	Chain-ID	Start	End
2V5O	A	1559	1566
2V5O	A	1706	1713
2V5O	A	1850	1856
2V5O	A	2039	2046
2V70	A	506	512
2V70	A	510	519
2V9S	A	273	279
2V9S	A	277	286
2VJ2	A	187	196
2VJ2	A	220	229
2VJ2	A	234	245
2VJ2	A	253	262
2VJ2	A	265	276
2VJ2	A	271	282
2VJ2	A	284	293
2VJ2	A	324	333
2VLW	A	46	57
2VLW	A	58	63
2VRP	A	5	16

Additional file 1: Table S3: Disulphide loops at the surface of PDB proteins.

PDB-ID	Chain-ID	Start	End
2VRP	B	2	13
2VT4	A	192	198
2VT4	B	192	198
2VTC	A	101	105
2W50	A	45	56
2W61	A	419	424
2W86	A	7	17
2W9O	A	4	13
2WBA	A	52	57
2WBA	B	52	57
2WFH	A	727	733
2WFH	A	731	740
2WFH	B	727	733
2WFH	B	731	740
2WFT	A	608	617
2WFT	A	612	623
2WFT	A	625	634
2WFT	A	639	649
2WFT	A	657	666

Additional file 1: Table S3: Disulphide loops at the surface of PDB proteins.

PDB-ID	Chain-ID	Start	End
2WFU	A	6	11
2WGC	A	35	40
2WGC	A	78	83
2WGC	A	121	126
2WGC	A	164	169
2WML	A	62	67
2WO3	A	108	118
2WPH	E	88	99
2WQZ	A	306	317
2WR7	A	473	477
2WR7	B	473	477
2WR7	C	473	477
2WRH	K	644	648
2X10	A	105	115
2X10	A	262	273
2X11	A	105	115
2X11	A	262	273
2X11	A	369	376
2X2U	A	137	142

Additional file 1: Table S3: Disulphide loops at the surface of PDB proteins.

PDB-ID	Chain-ID	Start	End
2XJH	A	4	9
2XOT	A	34	40
2XOT	A	38	47
2XOT	B	34	40
2XOT	B	38	47
2XR9	A	79	85
2XR9	A	123	129
2XR9	A	774	784
2XRC	A	25	36
2XRC	A	30	41
2XRC	A	168	178
2XWT	C	24	29
2XWT	C	31	41
2Y38	A	305	314
2Y38	A	329	338
2Y38	A	334	342
2Y38	A	364	373
2Y38	A	401	410
2YD0	A	736	743

Additional file 1: Table S3: Disulphide loops at the surface of PDB proteins.

PDB-ID	Chain-ID	Start	End
2YGO	A	182	192
2YGO	A	200	209
2YGQ	A	182	192
2YGQ	A	200	209
2YGQ	A	214	224
2YGQ	A	232	241
2YGQ	A	246	256
2YGQ	A	264	273
2YHF	A	71	82
2YIL	A	38	44
2YIL	A	82	86
2YIL	A	111	117
2YIL	B	38	44
2YIL	B	82	86
2YIL	B	111	117
2Z62	A	29	40
2Z63	A	29	40
2Z64	A	28	39
2Z64	C	95	105

Additional file 1: Table S3: Disulphide loops at the surface of PDB proteins.

PDB-ID	Chain-ID	Start	End
2Z66	A	24	30
2Z66	A	28	37
2Z7X	A	30	36
2Z7X	B	223	230
2Z81	A	30	36
3A2E	A	62	71
3A57	A	151	161
3A7Q	A	2133	2143
3A7Q	A	2151	2160
3A7Q	A	2415	2422
3A7Q	A	2482	2492
3A7Q	A	2486	2497
3A7Q	A	2499	2508
3AFC	A	107	117
3AFC	A	135	144
3AFC	B	107	117
3AFC	B	135	144
3AHQ	A	37	46
3ALQ	R	104	112

Additional file 1: Table S3: Disulphide loops at the surface of PDB proteins.

PDB-ID	Chain-ID	Start	End
3ALQ	S	104	112
3ALQ	T	104	112
3AOK	A	56	66
3AOK	A	71	77
3AOK	A	134	145
3AOK	A	149	158
3AOK	A	159	164
3ASI	A	1050	1061
3ASI	A	1072	1082
3B1B	A	194	198
3B2D	C	102	112
3B2D	D	102	112
3B3Q	A	342	353
3B3Q	B	342	353
3B4V	A	4	12
3B4V	B	4	12
3B4V	C	73	84
3B4V	C	145	156
3B4V	D	73	84

Additional file 1: Table S3: Disulphide loops at the surface of PDB proteins.

PDB-ID	Chain-ID	Start	End
3B4V	D	145	156
3B7E	A	124	129
3B7E	A	232	237
3B7E	A	280	289
3B7E	B	124	129
3B7E	B	232	237
3B7E	B	280	289
3BD9	A	294	304
3BDW	A	61	72
3BDW	B	119	130
3BG4	D	12	23
3BG4	D	17	28
3BI9	X	31	42
3BIW	A	342	353
3BIW	D	342	353
3BL8	A	317	328
3BL8	C	317	328
3BPN	B	9	19
3BPN	C	134	144

Additional file 1: Table S3: Disulphide loops at the surface of PDB proteins.

**PDB-ID**	**Chain-ID**	**Start**	**End**
3BT2	A	11	19
3BT2	A	33	42
3BT2	B	25	31
3BT2	U	6	12
3BT2	U	71	76
3BT2	U	98	105
3BT2	U	171	176
3BT2	U	197	205
3BT2	U	266	271
3BT4	A	68	79
3C05	A	21	27
3C05	B	21	27
3C9A	D	84	93
3CAD	A	145	150
3CFW	A	122	133
3CFW	A	144	153
3CIG	A	28	37
3CJ1	A	323	328
3CL4	A	307	312

Additional file 1: Table S3: Disulphide loops at the surface of PDB proteins.

PDB-ID	Chain-ID	Start	End
3CQL	A	97	105
3CU7	A	1520	1525
3CZU	A	105	115
3D12	A	493	503
3D12	A	565	574
3D12	D	493	503
3D12	D	565	574
3DCN	A	187	194
3DGC	R	71	79
3DI2	B	54	62
3DI2	B	88	98
3DIH	A	75	86
3DSL	A	162	167
3DSL	A	229	235
3DSL	A	253	259
3DSL	A	350	360
3DSL	A	403	414
3DSL	B	162	167
3DSL	B	229	235

Additional file 1: Table S3: Disulphide loops at the surface of PDB proteins.

PDB-ID	Chain-ID	Start	End
3DSL	B	253	259
3DSL	B	350	360
3DSL	B	403	414
3E0G	A	4	14
3E0G	A	31	39
3E0G	A	290	300
3ED3	A	87	96
3EDY	A	111	122
3EQA	A	286	294
3ETO	A	1686	1693
3EVS	C	21	25
3EVS	C	83	89
3EYJ	B	144	148
3F6K	A	415	425
3F8U	A	85	92
3FBX	A	147	157
3FBY	A	255	266
3FBY	A	282	287
3FF7	C	75	86

Additional file 1: Table S3: Disulphide loops at the surface of PDB proteins.

**PDB-ID**	**Chain-ID**	**Start**	**End**
3FF7	D	75	86
3FF8	C	75	86
3FIQ	A	44	48
3FKU	B	144	148
3FKU	D	144	148
3FKU	F	144	148
3FKU	X	104	109
3FKU	Y	104	109
3FKU	Z	104	109
3FRP	B	1463	1468
3FRP	B	1594	1603
3FV3	B	47	53
3FXI	A	29	40
3FXI	B	29	40
3FXI	C	95	105
3FXI	D	95	105
3G5C	A	394	401
3G5C	A	460	466
3G5C	A	485	491

Additional file 1: Table S3: Disulphide loops at the surface of PDB proteins.

**PDB-ID**	**Chain-ID**	**Start**	**End**
3G5C	A	583	593
3G5C	A	657	668
3G5C	A	702	711
3G8G	A	75	86
3G8H	A	75	86
3G9V	A	78	86
3GBO	A	157	162
3GBS	A	177	184
3GHM	A	360	371
3GHM	A	545	555
3GIS	X	351	360
3GIS	X	390	395
3GIS	X	399	407
3GIS	X	427	437
3GNO	A	197	205
3GRO	A	152	160
3GRO	B	152	160
3H53	A	42	49
3H53	B	42	49

Additional file 1: Table S3: Disulphide loops at the surface of PDB proteins.

PDB-ID	Chain-ID	Start	End
3H5C	B	51	62
3H5C	B	73	82
3HH2	A	6	16
3HH2	B	6	16
3HH2	C	66	77
3HH2	C	139	150
3HH2	C	216	227
3HH2	D	66	77
3HH2	D	139	150
3HH2	D	216	227
3HH7	A	58	63
3HH7	B	58	63
3HHC	A	155	162
3HHC	C	155	162
3HI7	A	177	181
3HI7	B	177	181
3HN4	A	74	84
3HTC	I	6	14
3HTC	I	6	16

Additional file 1: Table S3: Disulphide loops at the surface of PDB proteins.

PDB-ID	Chain-ID	Start	End
3HTC	I	14	22
3HTC	I	16	22
3HTC	I	22	28
3HTC	I	28	39
3IXY	A	105	116
3IXY	A	116	121
3IXY	B	105	116
3IXY	B	116	121
3IXY	C	105	116
3IXY	C	116	121
3IXY	D	34	45
3IXY	D	45	53
3IXY	E	34	45
3IXY	E	45	53
3IXY	F	34	45
3IXY	F	45	53
3IYG	D	389	393
3IYG	E	412	423
3IYG	G	354	360

Additional file 1: Table S3: Disulphide loops at the surface of PDB proteins.

PDB-ID	Chain-ID	Start	End
3IYG	H	355	361
3IYG	Z	491	500
3J0C	B	22	27
3J0C	E	22	27
3J0C	H	22	27
3J0C	K	22	27
3JVF	C	12	19
3JVF	C	154	165
3JVF	C	259	263
3JVF	C	263	272
3K51	B	115	126
3K6S	A	50	57
3K6S	A	476	487
3K6S	A	752	758
3K6S	A	1003	1008
3K6S	B	169	176
3K6S	B	437	448
3K6S	B	450	459
3K6S	B	484	495

Additional file 1: Table S3: Disulphide loops at the surface of PDB proteins.

PDB-ID	Chain-ID	Start	End
3K6S	B	542	551
3K6S	B	568	579
3K6S	B	581	590
3KAA	A	33	44
3KJ4	A	27	33
3KS9	A	432	439
3KS9	B	432	439
3KTZ	A	44	50
3L5H	A	112	122
3L5H	A	150	160
3L5H	A	436	444
3LAD	A	48	53
3LAD	B	48	53
3LAQ	A	12	20
3LAQ	A	34	43
3LAQ	U	6	12
3LAQ	U	71	76
3LAQ	U	99	106
3LAQ	U	169	174

Additional file 1: Table S3: Disulphide loops at the surface of PDB proteins.

**PDB-ID**	**Chain-ID**	**Start**	**End**
3LAQ	U	195	203
3LAQ	U	265	270
3LB6	C	145	155
3LMK	A	419	426
3LMK	B	419	426
3LPO	A	602	613
3LQM	A	66	74
3LRK	A	223	230
3M1C	B	149	160
3M7O	A	102	112
3M9Z	A	94	105
3MAW	A	338	347
3MAW	A	362	370
3MAW	A	394	399
3MC2	A	166	177
3MQ4	A	430	437
3MTX	A	97	107
3MUU	A	19	25
3MUU	A	428	438

Additional file 1: Table S3: Disulphide loops at the surface of PDB proteins.

PDB-ID	Chain-ID	Start	End
3MUU	B	19	25
3MUU	B	428	438
3MUU	C	19	25
3MUU	C	428	438
3MUW	A	62	68
3MUW	A	63	68
3MUW	A	68	78
3MUW	A	301	306
3MUW	A	370	376
3MUW	A	370	380
3MUW	A	376	380
3MUW	D	62	68
3MUW	D	63	68
3MUW	D	68	78
3MUW	D	301	306
3MUW	D	370	376
3MUW	D	370	380
3MUW	D	376	380
3MUW	E	62	68

Additional file 1: Table S3: Disulphide loops at the surface of PDB proteins.

PDB-ID	Chain-ID	Start	End
3MUW	E	63	68
3MUW	E	68	78
3MUW	E	301	306
3MUW	E	370	376
3MUW	E	370	380
3MUW	E	376	380
3MUW	F	62	68
3MUW	F	63	68
3MUW	F	68	78
3MUW	F	301	306
3MUW	F	370	376
3MUW	F	370	380
3MUW	F	376	380
3MUW	U	16	25
3MUW	U	19	25
3MUW	X	16	25
3MUW	X	19	25
3MUW	Y	16	25
3MUW	Y	19	25

Additional file 1: Table S3: Disulphide loops at the surface of PDB proteins.

PDB-ID	Chain-ID	Start	End
3MUW	Z	16	25
3MUW	Z	19	25
3MYW	I	18	26
3MYW	I	36	43
3MYW	I	45	53
3NK3	A	334	340
3NK3	B	334	340
3NKM	A	79	85
3NKM	A	123	129
3NKM	A	770	780
3NSJ	A	396	406
3NSJ	A	524	533
3NVN	A	77	86
3NVQ	A	120	126
3NVQ	A	143	152
3NVQ	A	587	596
3NVQ	E	120	126
3NVQ	E	143	152
3NVQ	E	587	596

Additional file 1: Table S3: Disulphide loops at the surface of PDB proteins.

PDB-ID	Chain-ID	Start	End
3OG4	B	56	64
3OJY	A	489	498
3OJY	B	68	79
3OJY	B	471	480
3P1I	A	116	126
3P2T	A	133	143
3P9D	B	335	340
3P9D	B	359	365
3P9D	D	275	283
3P9D	D	399	403
3P9D	G	368	374
3P9D	H	42	49
3P9D	J	335	340
3P9D	J	359	365
3P9D	L	275	283
3P9D	L	399	403
3P9D	O	368	374
3P9D	P	42	49
3PLC	A	44	55

Additional file 1: Table S3: Disulphide loops at the surface of PDB proteins.

PDB-ID	Chain-ID	Start	End
3PLC	A	56	61
3POY	A	703	714
3POY	A	725	735
3POY	A	906	914
3POY	A	1110	1121
3POY	A	1132	1142
3PRX	B	1485	1490
3PRX	B	1616	1625
3QCW	A	687	698
3QCW	A	709	719
3QCW	A	890	898
3QCW	A	1094	1105
3QCW	A	1116	1126
3QIB	D	69	75
3QO4	A	133	144
3QS7	E	103	114
3QS7	E	232	241
3QS7	E	381	392
3QS7	F	103	114

Additional file 1: Table S3: Disulphide loops at the surface of PDB proteins.

PDB-ID	Chain-ID	Start	End
3QS7	F	232	241
3QS7	F	381	392
3R0L	B	75	86
3R0L	D	75	86
3RJR	A	256	265
3RJR	B	256	265
3RKI	A	322	333
3RKI	A	358	367
3RKI	A	382	393
3RKI	A	416	422
3RKI	B	322	333
3RKI	B	358	367
3RKI	B	382	393
3RKI	B	416	422
3RKI	C	322	333
3RKI	C	358	367
3RKI	C	382	393
3RKI	C	416	422
3RLG	A	51	57

Additional file 1: Table S3: Disulphide loops at the surface of PDB proteins.

**PDB-ID**	**Chain-ID**	**Start**	**End**
3RRC	A	230	237
3RRC	B	230	237
3S2K	A	893	904
3S2K	A	1207	1218
3S2K	B	893	904
3S2K	B	1207	1218
3S8J	A	142	153
3S94	A	286	297
3S94	A	592	603
3S98	A	52	60
3S98	A	256	264
3SDY	B	144	148
3SE4	A	52	60
3SE4	A	256	264
3SE4	C	58	66
3TBD	A	52	60
3TBD	A	270	279
3TBD	A	290	299
3TGL	A	235	244

Additional file 1: Table S3: Disulphide loops at the surface of PDB proteins.

PDB-ID	Chain-ID	Start	End
3TON	A	1557	1568
3ZYO	A	59	65
3ZYO	A	63	74
4SBV	A	169	176
4SBV	B	169	176
4SBV	C	169	176
6RLX	A	6	11
6RLX	C	6	11
7MDH	A	24	29
7MDH	B	24	29

Additional file 1: Table S4: Disulphide loops at the interface of PDB proteins.

PDB-ID	Chain-ID	Start	End
PDB-ID	CHAIN-ID	START	END
1A22	B	238	248
1AN3	A	180	188

Additional file 1: Table S4: Disulphide loops at the interface of PDB proteins.

PDB-ID	Chain-ID	Start	End
1AN3	B	14	24
1AN3	C	14	24
1APH	A	6	11
1APY	A	41	46
1APY	C	41	46
1ATJ	A	44	49
1ATJ	D	44	49
1ATJ	E	44	49
1ATJ	F	44	49
1AUT	L	98	109
1B17	A	6	11
1B1Z	A	87	98
1B1Z	B	87	98
1B1Z	C	87	98
1B1Z	D	87	98
1BCP	B	192	199
1BCP	D	103	109
1BCP	F	92	98
1BHT	A	74	84

Additional file 1: Table S4: Disulphide loops at the interface of PDB proteins.

**PDB-ID**	**Chain-ID**	**Start**	**End**
1BP3	B	212	222
1C3A	A	4	15
1C3A	B	204	215
1C5M	D	22	27
1C5M	F	1	12
1CDT	A	42	53
1CDT	A	54	59
1CDT	B	42	53
1CDT	B	54	59
1CN4	A	28	38
1CQE	A	59	69
1CQE	B	59	69
1CSB	B	108	119
1CVU	A	59	69
1CVU	B	2059	2069
1CVW	L	91	102
1DN2	E	2	12
1DN2	F	2	12
1DX5	I	399	407

Additional file 1: Table S4: Disulphide loops at the interface of PDB proteins.

**PDB-ID**	**Chain-ID**	**Start**	**End**
1DX5	I	427	437
1EDM	B	51	62
1EDM	C	51	62
1EDM	C	73	82
1ES7	B	240	244
1ETH	C	286	297
1EXT	A	139	150
1EXT	A	156	162
1EXT	B	139	150
1F45	A	109	120
1F6F	A	7	14
1F6F	A	192	197
1F6F	C	12	22
1FL7	B	87	94
1FLC	A	332	338
1FLC	C	332	338
1FLC	E	332	338
1G1Q	C	144	153
1G1Q	D	122	133

Additional file 1: Table S4: Disulphide loops at the interface of PDB proteins.

**PDB-ID**	**Chain-ID**	**Start**	**End**
1G1Q	D	144	153
1G72	B	6	12
1G72	D	6	12
1G9I	I	309	317
1GH7	A	11	21
1GH7	A	226	236
1GH7	B	11	21
1GH7	B	226	236
1GL0	I	17	27
1GL1	I	17	28
1GPQ	A	57	62
1GPQ	B	57	62
1H4I	B	6	12
1H4I	D	6	12
1HC9	A	29	33
1HCN	B	93	100
1HDM	B	25	35
1HSL	A	38	45
1HSL	B	38	45

Additional file 1: Table S4: Disulphide loops at the interface of PDB proteins.

PDB-ID	Chain-ID	Start	End
1HYR	A	96	105
1HYR	A	99	110
1HYR	B	96	105
1HYR	B	99	110
1IAR	B	9	19
1ICF	I	227	234
1ILL	R	4	14
1ILM	B	10	20
1ING	A	124	129
1ING	B	124	129
1IOD	G	417	422
1ISF	A	239	248
1ISF	B	239	248
1IVO	C	33	42
1IVO	D	33	42
1IVY	A	213	218
1IVY	B	213	218
1J34	C	418	423
1JK4	A	74	79

Additional file 1: Table S4: Disulphide loops at the interface of PDB proteins.

PDB-ID	Chain-ID	Start	End
1JK4	B	1	6
1JV2	B	588	598
1JZN	A	3	14
1JZN	B	3	14
1JZN	C	3	14
1JZN	D	3	14
1JZN	E	3	14
1K7T	A	35	40
1K7T	A	78	83
1K8I	B	24	34
1K9I	A	256	267
1K9I	C	256	267
1K9I	D	256	267
1K9I	E	256	267
1K9I	F	256	267
1K9I	I	256	267
1K9I	J	256	267
1KBA	A	59	64
1KBA	B	27	31

Additional file 1: Table S4: Disulphide loops at the interface of PDB proteins.

PDB-ID	Chain-ID	Start	End
1KBA	B	59	64
1KCG	A	96	105
1KCG	A	99	110
1KCG	B	96	105
1KCG	B	99	110
1KIG	L	389	400
1KTZ	B	115	120
1LBE	A	239	248
1LBE	B	239	248
1LI1	A	65	71
1LI1	A	176	182
1LI1	B	65	71
1LI1	B	176	182
1LI1	C	65	71
1LI1	C	176	182
1LI1	D	65	71
1LI1	D	176	182
1LI1	E	65	71
1LI1	E	176	182

Additional file 1: Table S4: Disulphide loops at the interface of PDB proteins.

PDB-ID	Chain-ID	Start	End
1LI1	F	65	71
1LI1	F	176	182
1LK1	C	29	33
1LK1	C	48	59
1LK1	F	29	33
1LK1	F	48	59
1LK9	A	41	50
1LK9	B	41	50
1LNL	A	47	58
1LRW	B	6	12
1LRW	D	6	12
1LYA	A	46	53
1LYA	C	46	53
1M3D	A	65	71
1M3D	A	176	182
1M3D	B	65	71
1M3D	B	176	182
1M3D	C	64	70
1M3D	C	173	180

Additional file 1: Table S4: Disulphide loops at the interface of PDB proteins.

**PDB-ID**	**Chain-ID**	**Start**	**End**
1M3D	D	65	71
1M3D	D	176	182
1M3D	E	65	71
1M3D	E	176	182
1M3D	F	64	70
1M3D	F	173	180
1MCT	I	16	27
1MHL	C	221	232
1MHL	D	221	232
1MOX	B	191	199
1MOX	C	34	43
1MOX	D	34	43
1MX1	A	1274	1285
1MX1	B	2274	2285
1MX1	C	3274	3285
1MX1	D	4274	4285
1MX1	E	5274	5285
1MX1	F	6274	6285
1N69	B	36	47

Additional file 1: Table S4: Disulphide loops at the interface of PDB proteins.

**PDB-ID**	**Chain-ID**	**Start**	**End**
1N8Z	C	565	574
1OB1	C	7	18
1OB1	C	30	41
1OC0	B	25	31
1OQE	K	9	20
1OQE	L	9	20
1OQE	M	9	20
1OQE	N	9	20
1OQE	O	9	20
1OQE	P	9	20
1OQE	Q	9	20
1OQE	R	9	20
1PBI	A	14	22
1PFX	L	88	99
1QFX	A	394	402
1QFX	B	394	402
1QKD	A	43	54
1QKD	A	55	60
1QKD	B	43	54

Additional file 1: Table S4: Disulphide loops at the interface of PDB proteins.

PDB-ID	Chain-ID	Start	End
1QKD	B	55	60
1QO3	C	145	150
1R46	A	56	63
1R46	B	56	63
1RD8	B	144	148
1RD8	D	144	148
1RD8	F	144	148
1RFN	B	88	99
1RLX	A	6	11
1RUY	I	644	648
1RUY	K	644	648
1RUY	M	644	648
1T7H	A	5	13
1T7H	B	5	13
1TAB	I	24	32
1ULK	A	36	40
1ULK	A	77	81
1UMR	A	4	15
1UMR	C	204	215

Additional file 1: Table S4: Disulphide loops at the interface of PDB proteins.

PDB-ID	Chain-ID	Start	End
1UUZ	A	59	64
1V0Z	A	130	135
1V0Z	C	130	135
1V0Z	D	130	135
1V4L	A	4	15
1V4L	B	204	215
1V4L	C	4	15
1V4L	D	204	215
1V4L	E	4	15
1V4L	F	204	215
1W1C	A	59	64
1W1C	B	59	64
1W1E	A	61	66
1W1E	B	61	66
1WUW	A	16	25
1WUW	B	66	75
1XDT	R	134	143
1XDT	T	461	471
1XG2	B	9	18

Additional file 1: Table S4: Disulphide loops at the interface of PDB proteins.

PDB-ID	Chain-ID	Start	End
1XTA	A	167	174
1XTA	A	170	179
1XTA	B	167	174
1XTA	B	170	179
1XU1	R	89	100
1XU1	R	93	104
1XU1	S	89	100
1XU1	S	93	104
1XU1	T	89	100
1XU1	T	93	104
1XWD	B	87	94
1Y4M	A	86	93
1Y4M	B	86	93
1Y4M	C	86	93
1YPO	A	144	155
1YPO	B	144	155
1YPQ	A	144	155
1YPQ	B	144	155
1YUK	B	437	448

Additional file 1: Table S4: Disulphide loops at the interface of PDB proteins.

PDB-ID	Chain-ID	Start	End
1YUK	B	450	459
1YWH	A	6	12
1YWH	A	71	76
1YWH	A	98	105
1YWH	C	6	12
1YWH	C	98	105
1YWH	E	6	12
1YWH	E	71	76
1YWH	G	6	12
1YWH	G	98	105
1YWH	I	6	12
1YWH	I	98	105
1YWH	K	6	12
1YWH	K	98	105
1YWH	M	6	12
1YWH	M	98	105
1YWH	O	6	12
1YWH	O	71	76
1ZEI	C	38	43

Additional file 1: Table S4: Disulphide loops at the interface of PDB proteins.

PDB-ID	Chain-ID	Start	End
1ZEI	D	38	43
2ATY	A	154	159
2ATY	A	154	161
2ATY	A	156	161
2ATY	B	154	159
2ATY	B	154	161
2AW2	B	4	15
2B9B	A	324	333
2BPD	A	119	130
2BPD	B	119	130
2COV	D	413	418
2COV	E	413	418
2COV	F	413	418
2COV	G	413	418
2COV	H	413	418
2COV	I	413	418
2DRF	B	279	289
2DTG	E	228	237
2E3X	B	2	13

Additional file 1: Table S4: Disulphide loops at the interface of PDB proteins.

PDB-ID	Chain-ID	Start	End
2F2L	A	390	396
2FT3	A	27	33
2FT3	B	27	33
2G81	I	24	32
2GIY	A	314	323
2H62	C	40	44
2H7Z	A	55	66
2H7Z	B	13	21
2HEV	R	31	42
2OTP	A	132	142
2OTP	B	132	142
2QBX	A	105	115
2QC1	A	29	33
2QTS	A	173	180
2QTS	B	173	180
2QTS	C	173	180
2R0R	A	36	47
2R0R	B	36	47
2R6P	C	105	116

Additional file 1: Table S4: Disulphide loops at the interface of PDB proteins.

PDB-ID	Chain-ID	Start	End
2V5E	A	161	167
2WBA	A	52	57
2WBA	B	52	57
2WFU	A	6	11
2WPH	E	88	99
2WRH	K	644	648
2XOT	A	34	40
2XOT	A	38	47
2XOT	B	38	47
2XWT	C	31	41
2YIL	A	82	86
2YIL	B	82	86
2Z64	C	95	105
3ALQ	T	104	112
3B2D	C	102	112
3B2D	D	102	112
3BDW	A	61	72
3BDW	B	119	130
3BPN	B	9	19

Additional file 1: Table S4: Disulphide loops at the interface of PDB proteins.

PDB-ID	Chain-ID	Start	End
3BT2	A	33	42
3BT2	B	25	31
3BT2	U	6	12
3BT2	U	266	271
3C9A	D	84	93
3EVS	C	21	25
3FF8	C	75	86
3FKU	D	144	148
3FKU	Z	104	109
3FXI	C	95	105
3FXI	D	95	105
3GIS	X	399	407
3GIS	X	427	437
3GRO	B	152	160
3HH2	A	6	16
3HH2	B	6	16
3HH2	C	216	227
3HH7	A	58	63
3HH7	B	58	63

Additional file 1: Table S4: Disulphide loops at the interface of PDB proteins.

**PDB-ID**	**Chain-ID**	**Start**	**End**
3IXY	C	105	116
3JVF	C	259	263
3JVF	C	263	272
3K6S	A	476	487
3K6S	B	169	176
3K6S	B	581	590
3LAQ	A	34	43
3LAQ	U	6	12
3M1C	B	149	160
3MYW	I	18	26
3MYW	I	36	43
3MYW	I	45	53
3NVQ	A	587	596
3NVQ	E	587	596
3R0L	B	75	86
6RLX	A	6	11
6RLX	C	6	11

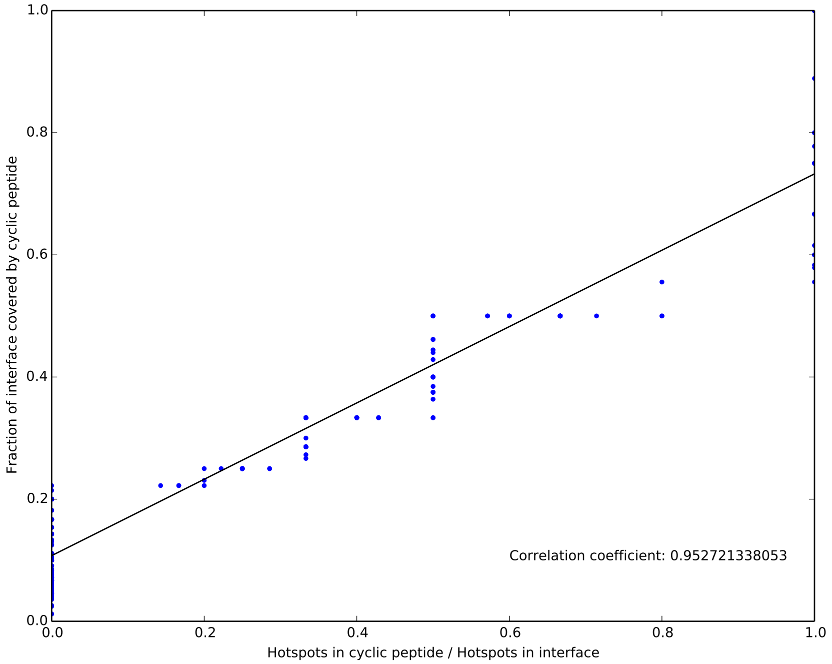

Additional file 1: Figure S1: The proportion of protein-protein interface hotspots contained in a cyclic peptide is well correlated with the fraction of the interface residues contained in the cyclic peptide.

Additional file 1: Table S1: *De-novo* disulphide loop structure model accuracy vs. X-ray crystal structures. Five PEP-FOLD models were calculated for each PDB loop sequence, with model1 being the lowest energy, and model5 being the highest energy. The CKTNGDC sequence appears twice it appears chain I and chain U of PDB ID 3BT2, and both loops interact with different proteins.

Loop Sequence	PEP-FOLD model	RMSD
CVVGYIGERC	model1	2.374
CVVGYIGERC	model2	3.601
CVVGYIGERC	model3	3.077
CVVGYIGERC	model4	3.66
CVVGYIGERC	model5	3.122
CKTNGDC	model1	2.319
CKTNGDC	model2	2.714
CKTNGDC	model3	1.651
CKTNGDC	model4	1.395
CKTNGDC	model5	2.6
CKTNGDC-2	model1	2.393
CKTNGDC-2	model2	2.571
CKTNGDC-2	model3	1.937
CKTNGDC-2	model4	1.878
CKTNGDC-2	model5	2.659
CTKSIPPQC	model1	1.532
CTKSIPPQC	model2	2.466
CTKSIPPQC	model3	2.882
CTKSIPPQC	model4	2.669
CTKSIPPQC	model5	2.156
CSYYQSC	model1	2.033
CSYYQSC	model2	2.707
CSYYQSC	model3	2.704
CSYYQSC	model4	2.661
CSYYQSC	model5	2.662
CKPHDC	model1	1.892
CKPHDC	model2	1.093
CKPHDC	model3	1.265
CKPHDC	model4	1.528
CKPHDC	model5	1.502
CTKSMPPKC	model1	3.358
CTKSMPPKC	model2	3.075
CTKSMPPKC	model3	1.844
CTKSMPPKC	model4	1.822
CTKSMPPKC	model5	2.301
CPTAGPNERVKC	model1	3.845
CPTAGPNERVKC	model2	3.69
CPTAGPNERVKC	model3	3.583
CPTAGPNERVKC	model4	3.467
CPTAGPNERVKC	model5	3.566

Additional file 1: Table S2: Human proteins interacting with virus proteins.

**Virus Accession**	**Virus peptide sequence**	**Virus Protein Names**	**Human interactor accession**	**Human interactors name**
Q1HVB5	CELGWC	Uncharacterized protein BNLF2b: Human herpesvirus 4	P28799	GRN: Granulins
O41974	CKRRC	Immediate-early protein, 73: Murid herpesvirus 4	Q04206	RELA: Transcription factor p65
P03227	CRRPC	Single stranded DNA-binding protein: Human herpesvirus 4	Q9UBX5, Q15723	ELF2: ETS-related transcription factor Elf-2, FBLN5 Fibulin-5 Developmental arteries and neural crest EGF-like protein
P03254	CNSSTDSC	Early E1A 32 kDa protein: Human adenovirus 2	P62826, P20226	TBP: TATA-box-binding protein transcription factor, RAN: GTP-binding nuclear protein Ran
Q77PU6	CTIRSDC	Protein U90: Human herpesvirus 6B	P63165	SUMO1: Small ubiquitin-related modifier 1

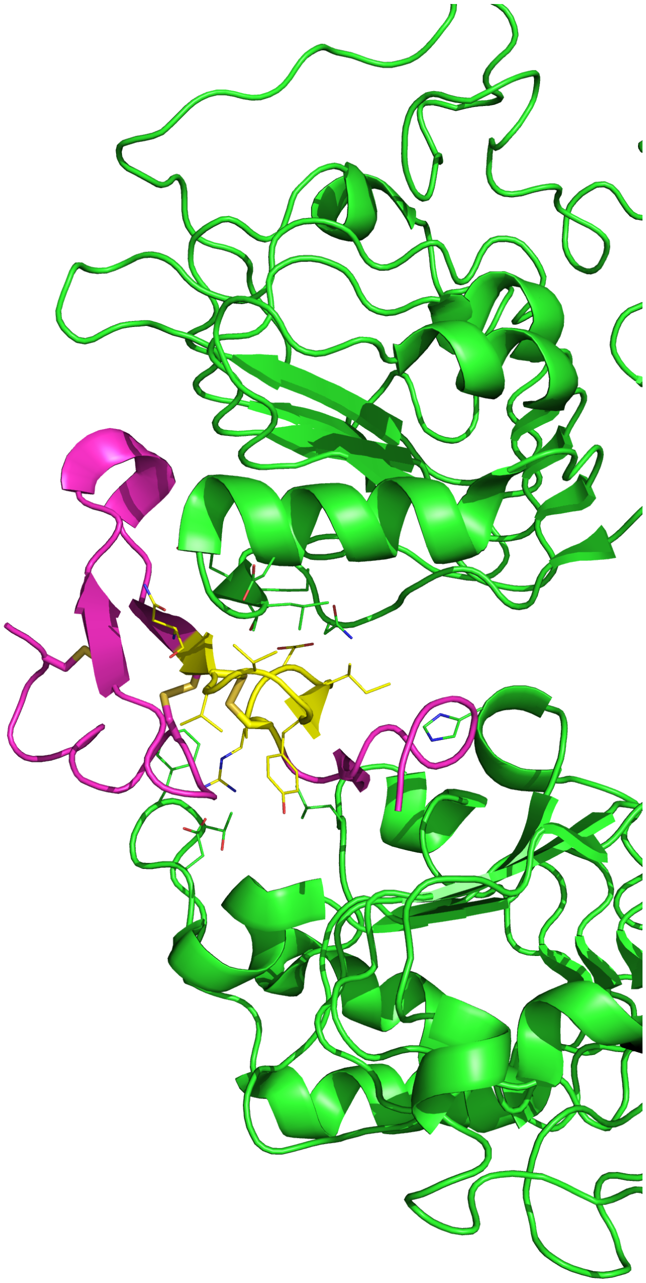

Additional file 1: Figure S2: Three-dimensional structure of the EGF-EGFR interaction. The EGF receptor is in green and EGF is in pink, with the CVVGYIGERC cyclic peptide segment of EGF highlighted in yellow. The cyclic peptide side chains are shown, along with interacting EGFR side chains (those within 4Å of the cyclic peptide).

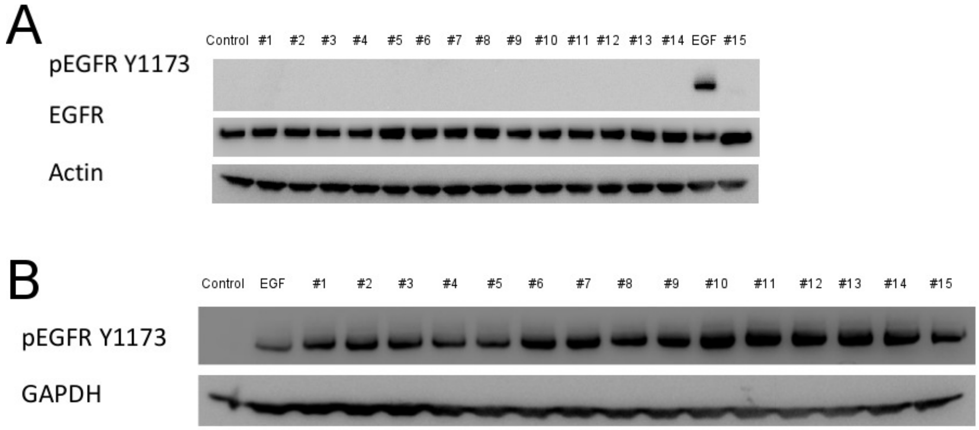

Additional file 1: Figure S3: EGF derived cyclic peptides measured EGF Receptor activation and competitive binding to EGFR with native EGF. (a) EGF receptor activation (revealed by phosphorylation of Tyrosine 1173) with total EGFR and Actin levels. Numbers indicate cyclic peptides. (b) EGF receptor activation after competitive binding of EGF and cyclic peptide to the EGF receptor.
